# On the Involvement of Copper Binding to the N-Terminus of the Amyloid Beta Peptide of Alzheimer's Disease: A Computational Study on Model Systems

**DOI:** 10.4061/2011/539762

**Published:** 2011-12-01

**Authors:** Samira Azimi, Arvi Rauk

**Affiliations:** Department of Chemistry, The University of Calgary, Calgary AB, Canada T2N 1N4

## Abstract

Density functional and second order Moller-Plesset perturbation theoretical methods, coupled with a polarizable continuum model of water, were applied to determine the structures, binding affinities, and reduction potentials of Cu(II) and Cu(I) bound to models of the Asp1, Ala2, His6, and His13His14 regions of the amyloid beta peptide of Alzheimer's disease. The results indicate that the N-terminal Asp binds to Cu(II) together with His6 and either His13 or His14 to form the lower pH Component I of A*β*. Component II of A*β* is the complex between Cu(II) and His6, His13, and His14, to which an amide O (of Ala2) is also coordinated. Asp1 does not bind to Cu(II) if three His residues are attached nor to any Cu(I) species to which one or more His residues are bound. The most stable Cu(I) species is one in which Cu(I) bridges the N_*δ*_ of His13 and His14 in a linear fashion. Cu(I) binds more strongly to A*β* than does Cu(II). The computed reduction potential that closely matches the experimental value for Cu(II)/A*β* corresponds to reduction of Component II (without Ala2) to the Cu(I) complex after endergonic attachment of His6.

## 1. Introduction

The sequence of the human amyloid beta peptide, A*β*(1-42), in single-letter code, is:

D_1_AGFRH_6_DSGY_10_EVH_13_H_14_QKLVFFAED-VGSNKGAIIGLM_35_VGGVVIA_42_

Cupric ion (Cu^2+^) forms a 1 : 1 complex with A*β*(1-40) or A*β*(1-42) [1] with approximately picomolar or nanomolar affinity (*K*_*d*_^cond^ = 1 × 10^−11^ M for A*β*(1-42); [1] *K*_*d*_^cond^ = 57 ± 5 × 10^−9^ M for A*β*(1-40) [2]). The presence of added or *in vivo* buffers may lower the effective affinity to a considerable extent [1, 3]. The Cu(II) is bound as type 2, that is, distorted square planar arrangements of ligands with possibly one or two additional axial ligands. EPR measurements indicate a 3N1O equatorial coordination pattern [4]. At room temperature, two [3, 5–7] or three [8] forms whose populations are dependent on pH are observed. The lower pH component is referred to as component I (or Ia and Ib) [9] while the higher pH component is Component II, all with 3N1O coordination. It is likely that the three N ligands are due to a combination of the imidazole groups of His6, His13, His14, a deprotonated amide N, and the N-terminus since these are the most common ligands for copper bound in proteins. Indeed, there is direct evidence, obtained by ESR experiments on A*β*(1-16) in which the histidines were isotopically enriched with ^15^N, that all three His residues bind to Cu(II) at physiological pH = 7.4 [10–12]. The origin of the O ligand is a subject of some debate, although Tyr10, Glu3, Asp7, and Glu11 have been ruled out, as has water [13]. On the basis of hyperfine sublevel correlation (HYSCORE) spectroscopy applied to site-specific ^13^C and ^15^N labeled A*β*(1-16), the carbonyl of Ala2 is implicated as the O ligand in the coordination mode at higher pH = 8.0 (Component II), along with all three His residues [12]. This result is supported by computational modeling of A*β*(1-16) [14]. At higher pH (pH = 8.7–9), studies by both EPR [6] and NMR [7] on labeled compounds seem to indicate that deprotonation and coordination of the amide N of Ala2 occurs, together with the N-terminal NH_2_ and one or two His side chains. Hureau and Faller and coworkers [6, 7] assigned this high pH structure as “Component II,” but it is at odds with the structure deduced by others at physiological pH that does not involve the N-terminus and was designated Component II.

There is general but not universal agreement that the N-terminus is also part of the native A*β*/Cu binding site, although the nature of its involvement is still unclear. In an earlier study, Karr and coworkers found that copper binding to A*β* was sensitive to changes in the N-terminus, including deletion [4], but, in a subsequent study, they note that removal or mutation of Asp1 does *not* disrupt the equatorial coordination sphere [13]. They propose that the N-terminus participates via hydrogen bonding to an axial ligand [13]. Kowalik-Jankowska et al. carried out potentiometric and spectroscopic measurements on both human and mouse A*β*(1-16) and A*β*(1-28) over a wide pH range, 2.5–10.5, and noted a significant shift in the coordination pattern upon acetylation of the N-terminus in all cases [15]. Barnham and coworkers used multifrequency CW-EPR spectroscopy applied to site-specific ^15^N-labeling at Asp1, His6, His13, and His14 of A*β*(1-16) to deduce the presence at pH = 6-7 of two independent 3N1O Cu^2+^ coordination modes both of which incorporated the N-terminal NH_2_ group, an O atom, His6, but only one of His13 (Component Ia) or His14 (Component Ib) [9]. On the other hand, Hong et al., propose that the 3N1O coordination arises from an equilibrium between *three* structures at pH = 6.5–7.4 that all incorporate, beside an “O” atom, the N-terminal NH_2_ group, His6, and either a deprotonated backbone amide residue or one of His13 or His14, but not simultaneously His13 and His14 [8]. Both low pH results are in qualitative agreement with the structure assigned to Component I at pH = 6 by Hureau and Faller and coworkers who proved coordination of the NH_2_ and carbonyl groups of Asp1 together with His6 and one of His13 and His14.

Besides being highly pH dependent, indications are that copper coordination is probably size dependent as well. NMR studies of Cu^2+^(aq) interacting with full-length uniformly ^15^N-labelled A*β*(1-40) at pH = 7.3 found that the Asp1 signals were not shifted upon addition of Cu^2+^ and seem to indicate that the N-terminus is not involved in copper binding at all [16].

On the basis of ab initio computations on model systems, we have previously proposed that the primary binding site of Cu(II) to A*β* is His13His14 which provide two of the three observed N ligands through N_*δ*_ of the imidazole groups. By our procedures, we could not verify the presence or absence of the carbonyl O atom of Ala2 as has been proposed experimentally. However, in the computations, the O of the intervening amide group (O of His13) inevitably became coordinated to the Cu(II) [17]. We proposed that this amide oxygen corresponds to the mysterious “O” of the 3N1O coordination pattern and that the third “N” was probably that of His6 or Asp1. Possibly because of the excessively high affinity constant calculated in that study, log⁡_10_⁡*K*_*aff*⁡_ = 19, this suggestion has not been taken seriously by any of the experimental groups that have examined Cu coordination to A*β*. We have since reevaluated the binding by the procedures employed in the present work and found a substantially lower value, log⁡_10_⁡*K*_*aff*⁡_ = 6.3, [18] that is more in accordance with the accepted data for Cu(II) binding to A*β* [2]. We note that isothermal calorimetry experiments by Hong et al. found that Cu^2+^ binding to His13His14 is most favored by the enthalpic contribution to the free energy change but that the enthalpic preference was overwhelmed by an unfavorable entropic term [8].

Less is known experimentally about the coordination of Cu(I) to A*β*. We have previously [19, 20] modeled the attachment of Cu(I) to the His13His14 region of A*β* by computational methodology similar to that employed in the present work. The computations suggest that Cu(I) is dicoordinated and bound to the two His sidechains via the proximal N atoms (N_*δ*_) in a linear fashion. Himes et al. have demonstrated such binding experimentally by EXAFS and XANES data and results carried out on copper(I) complexes of small HisHis peptides [21], and fragments of A*β* [22]. Such a structure was also found by XAS spectroscopy on A*β*(1-40) [23]. Apparently, Cu(I) binds to A*β*(1-16) with femtomolar affinity [24], much higher than does Cu(II). The linear two-coordinate complexes were also able to add a third imidazole ligand in a T-shaped configuration in a dynamic process that interchanges all three His residues [25]. Interestingly, the two-coordinate complexes were resistant to oxidation, but the three-coordinate complexes were redox active [26]. However, the Cu(I) complexes with HisHis containing fragments were able to produce H_2_O_2_ in the presence of O_2_ but without added reducing agents. The amount of H_2_O_2_ produced was independent of the presence of Tyr10 (a potential source of electrons) or other residues such as Asp1 or His6 which are potential binding sites for the copper [22]. 

The reduction potential of Cu(II)/A*β* has been investigated by several groups. Aqueous Cu^2+^ has a one-electron reduction potential *E*° = 0.17 V versus the standard hydrogen electrode (SHE). An early report that monomeric Cu(II)/A*β*(1-42) had an exceptionally high reduction potential *E*° ≈ 0.7 V versus SHE [27] has been discounted as due to oligomer formation. The consensus value is *E*°(“Cu(II)/A*β*”/“Cu(I)/A*β*”) *≈* 0.30 V–0.34 V [28, 29] which represents a modest elevation of the oxidizing power of the Cu(II) upon complexation to A*β*. Brzyska et al., find a concentration- and buffer-dependent reduction of the reduction potential upon addition of Cu(II) to monomeric A*β*(1-40), although, in the absence of buffer, their value is similar to the others [30]. As noted above, there is uncertainty as to the actual nature of the oxidized and reduced species involved. Guilloreau et al. report a wide difference between the reduction potential and oxidation potential of copper bound to A*β*(1-28), 0.33 V and 0.63 V, respectively [29]. This is taken as an indication of different geometries at the Cu(II) and Cu(I) binding sites [30]. 

**Figure 1:**
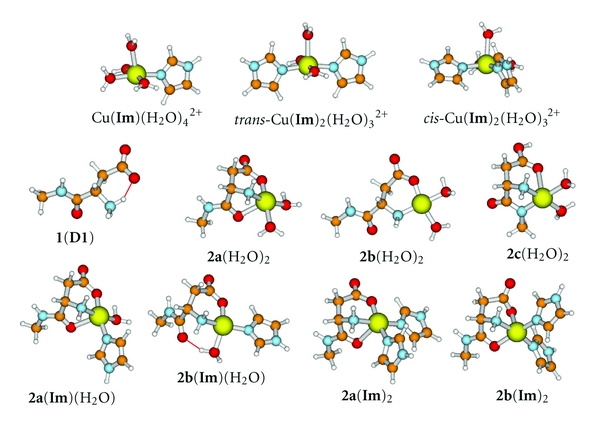
Structures with Cu(II), Asp1, and imidazole **Im**. Ball colors: Cu: large yellow; C: orange; N: blue; O: red; H: white.

**Figure 2:**
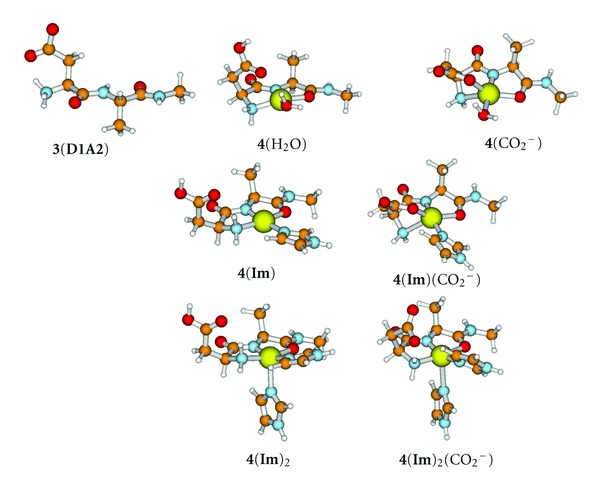
Structures with Cu(II), Asp1Ala2 **3**, and imidazole **Im**. Ball colors: Cu: large yellow; C: orange; N: blue; O: red; H: white.

**Figure 3:**
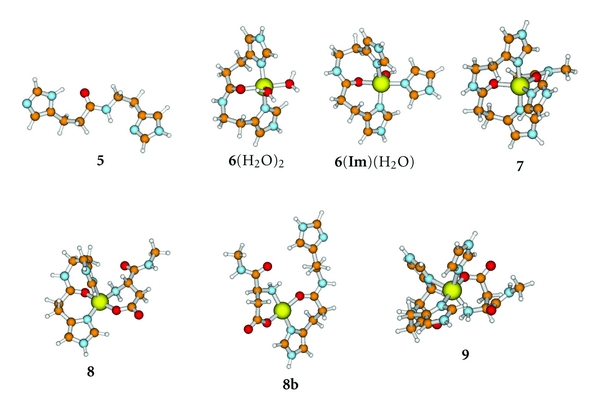
Structures with Cu(II), HisHis **5**, Asp1, and Imidazole **Im**. Ball colors: Cu = large yellow; C = orange; N = blue; O = red; H = white.

We have undertaken the present study in order to examine the role of Asp1 in the coordination of Cu(II) as well as Cu(I) in A*β*. We employ a higher level of theoretical treatment than was previously applied in order to estimate the binding affinities of complexes containing all combinations of Asp1, modeled by the N-methyl amide derivative, **1** (Figure 2), His6, modeled by imidazole (**Im**), an amide carbonyl, modeled by N-methylacetamide (NMA), and His13His14, modeled as previously [17, 20] by *N*-*α*-dihydrourocanylhistamine, **5** (Figure 3). Simultaneous involvement of both Asp1 and Ala2 were modeled by the N-methyl amide derivative of Asp1Ala2, **3** (Figure 1). Reduction potentials for various Cu(II)/Cu(I) couples were also derived and compared with experimental values in order to elucidate the nature of the species involved.

## 2. Computational Methods

All calculations were carried out with Gaussian 03 and 09 [31, 32] using the hybrid density functional method, B3LYP [33], and second-order Moller-Plesset perturbation theory. Gaseous-phase geometry optimization, harmonic frequency calculation, and thermochemical parameters were determined at the B3LYP/6-31 + G(d) basis set, which is henceforth referred to as the small basis set (SB). The frequency calculation confirmed that the optimized structures were at local minima on the potential energy hypersurface. The zero point energies were scaled by 0.9806 [34]. However, this was not done for the thermal correction of enthalpy or entropies. In the case of the zwitterionic Asp1, **1** (Figure 1) and Asp1Ala2, **3** (Figure 2), it was necessary to optimize the structure and carry out frequency analysis in the presence of the solvent reaction field (SCRF = IEFPCM), where IEFPCM is the integral equation following polarizable continuum model [35, 36] with the default parameters for water. Some structures had a great deal of conformational flexibility. Chemical intuition was used to seek the most stable structures, and no attempt was made to do a comprehensive search of conformational space. Instead, account was taken of conformational flexibility by the addition of an approximate entropy of mixing term, *R*ln⁡⁡(*n*), where *n* is an estimate of the number of conformers derived by simple rotamer counting [37]. Values of *n* are listed in Table S2 in Supplementary Materials available online at doi: 10.4061/2011/539762. Entropies were also converted to a state of 1 M by addition of the term for volume change, *R* ln⁡⁡(1/24.46), where 24.46 litres is the volume of 1 mol of ideal gas at 298 K. For more accurate enthalpies and to compensate for the lack of long-range dispersion energy in B3LYP, single-point energies were calculated at an approximation for the MP2/LB level,

(1) E(MP2/LB)≈E(MP2/SB)+E(B3LYP/LB)−E(B3LYP/SB),

where LB is the large basis set, 6-311+(2df,2p).

 Details of all computed quantities and structural information, are provided in Tables S1 and S2, respectively. Molden 4.0 was used as a visualization tool [38].

### 2.1. Free Energies of Solvation, Δ*G*_solv_, and Empirical Corrections

In order to calculate the free energy change in water, Δ*G*_(aq)_, the change in the free energy of solvation, ΔΔ*G*_solv_, was added to the free energy change in the gaseous phase, Δ*G*_(g)_, corrected for a standard state of 1 M. Δ*G*_solv_ was determined using IEFPCM [35, 36] as implemented in G03, and the B3LYP/SB density. In our experience, charged species are undersolvated by the IEFPCM with standard scaling of the united atom Hartree-Fock (UAHF) radii, so selective scaling was applied as follows: the radii of the metal ion and all atoms directly attached to it were scaled by a factor of 1.1; all other atoms were scaled by the default value, 1.2. Experimental rather than calculated relative free energies of solvation were applied where available in order to reduce errors further. For the proton, Δ*G*_solv_(H^+^) = −1107 kJ mol^−1^ was adopted [39]. The experimental value of Δ*G*_solv_ was adopted for H_2_O, −16.2 kJ mol^−1^, where the value reflects the fact that water is 55.6 M. For all other species, free energies of solvation were taken as calculated by the procedure described above.

### 2.2. Calculation of Reduction Potentials for “Cu(II)”/“Cu(I)” Redox Couples

The mechanisms proposed below involve only intermolecular single-electron transfer processes. The standard reduction potential of a “Cu(II)”/“Cu(I)” couple, relative to the standard hydrogen electrode (SHE), **E**°(“Cu(II)”/“Cu(I)”), is defined by

(2) $$E^{{}^{\circ}}\left(\text{“Cu}\left(\text{II}\right)\text{”}/\text{“Cu}\left(\text{I}\right)\text{”}\right)=-\left(\Delta G_{\left(\text{aq}\right)}^{\text{Cu}}-\Delta G_{\left(\text{aq}\right)}^{\text{SHE}}\right)/F,$$

where *F* is the Faraday constant, *F *= 96.485 kJ mol^−1^ V^−1^, Δ*G*_(aq)_^SHE^ is the free energy change for the standard hydrogen cell half reaction, (1/2)H_2(g)_ + e^−^ → H^+^_(aq)_, (Δ*G*_(aq)_^SHE^ = −418 kJ mol^−1^, ignoring the electron) [40], and Δ*G*_(aq)_^Cu^ is the calculated free energy change for reaction (3), again ignoring the electron

(3) $${\text{“Cu}\left(\text{II}\right)\text{”}}_{\left(\text{aq}\right)}+\;{\text{e}}^{-}\to \text{“}{\text{Cu}\left(\text{I}\right)\text{”}}_{\left(\text{aq}\right)}+a\text{L}.$$

In (3), “Cu(II)” and “Cu(I)” represent species containing oxidized and reduced copper, respectively. The symbol *a*L recognizes the fact that a number of ligands may be shed in the reduction process and that the associated entropy change may be an important component of the free energy change. The actual potential, *E*, of the half reaction under ambient conditions is related to the standard potential, *E*°, through the Nernst equation,

(4) $$E=E^{{}^{\circ}}-\left(\frac{RT}{F}\right)\ln Q,$$

where *Q* is the reaction quotient specifying concentrations of oxidized and reduced components and other species associated with the chemical change. In the special case that *n* protons are consumed in solution buffered at pH = 7 under otherwise standard conditions, the reaction quotient reduces to *Q* = 10^7*n*^, and the symbol, *E*°′, denotes the potential at pH 7 (*E*°′ = *E*° − (*RT*/*F*)ln⁡*Q* = *E*° − 0.41nV). 

For most of the energy differences calculated in the following sections, errors inherent in the calculation of absolute values could be expected to cancel yielding reliable relative energies. However, this is less likely to be the case for the calculation of aqueous free energy changes for reactions such as (3). Since a transition element is involved and the number of electrons changes, the enthalpy change will be less accurately described at this theoretical level than expected for lighter elements. An extreme case is illustrated by the difference between the calculated (at the B3LYP/LB level) and experimental second ionization potential of atomic copper (IE2 (calc) = 2008 kJ mol^−1^; IE2 (exp) = 1958 kJ mol^−1^ [41]). The discrepancy is most likely due to the unequal treatment of electron correlation (an enthalpic term). As in a previous publication [42], we assume that the error in the ionization potential of Cu^+^ will be present in the reduction potentials, *E*°(“Cu(II)”_(aq)_/“Cu(I)”_(aq)_), irrespective of the metal environment since they all involve the change in copper oxidation state from +2 to +1. Without correcting for the error in the enthalpy change, the calculated value for the reduction of aqueous cupric ion is *E*°(Cu(H_2_O)_5_^2+^/Cu(H_2_O)_3_^+^) = 0.42 V versus SHE, compared to the experimental value, *E*°(Cu^2+^(aq)/Cu^+^(aq)) = 0.17 V [41]. An empirical correction of +57 kJ mol^−1^ brings the calculated and experimental numbers into agreement. Thus for addition of an electron to any Cu^2+^ species, the correction to the calculated Δ*H* is taken as +57 kJ mol^−1^. 

With the procedures described above, we expect that aqueous free energy changes, Δ*G*_(aq)_, will be accurate to ±15 kJ mol^−1^ for all of the reactions considered.

## 3. Results and Discussion

The B3LYP/SB-optimized structures and all calculated energies and thermochemical properties are provided in Tables S1 and S2 and shown in Figures 1–3. Chemical transformations and aqueous free energy changes are given in the following in the form of numbered equations which are repeated in Table 1 along with a complete breakdown of the components at the MP2/LB level. The corresponding data at the B3LYP/LB level are given in Table S3.

## 4. Interaction of Cu^**2**+^(aq) with the N-terminal Asp1 **1**

The N-terminal Asp1 of A*β* is modeled by **1** (Figure 1), where the C-terminus is derivatized by NHCH_3_. The most stable form of **1** in water is zwitterionic. The most stable form of the aqueous cupric ion is the pentaaqua structure, Cu(H_2_O)_5_^2+^. Reaction with **1** yielded numerous aquated structures **2** with different patterns of chelation:

(5a) $$\begin{array}{cc} 1+\text{Cu}{{\left({\text{H}}_{2}\text{O}\right)}_{5}}^{2+}\to 2a{\left({\text{H}}_{2}\text{O}\right)}_{2}+3{\text{H}}_{2}\text{O}+{\text{H}}^{+}; & \\ \Delta G_{\left(5\text{a}\right)}=-18.0\;\text{kJ}\;{\text{mol}}^{-1}, & \end{array}$$

(5b) $$\begin{array}{cc} 1+\text{Cu}{{\left({\text{H}}_{2}\text{O}\right)}_{5}}^{2+}\to \;2b{\left({\text{H}}_{2}\text{O}\right)}_{2}+3{\text{H}}_{2}\text{O}+{\text{H}}^{+}; & \\ \Delta G_{\left(5\text{b}\right)}=+14.3\;\text{kJ}\;{\text{mol}}^{-1}, & \end{array}$$

(5c) $$\begin{array}{cc} 1+\text{Cu}{{\left({\text{H}}_{2}\text{O}\right)}_{5}}^{2+}\to 2c{\left({\text{H}}_{2}\text{O}\right)}_{2}+3{\text{H}}_{2}\text{O}+{\text{H}}^{+};\; & \\ \Delta G_{\left(5\text{c}\right)}=+29.1\;\text{kJ}\;{\text{mol}}^{-1}, & \end{array}$$

where Δ*G*_(5a)_, Δ*G*_(5b)_, and Δ*G*_(5c)_, have been adjusted to pH = 7. Structures of **2a**(H_2_O)_2_, **2b**(H_2_O)_2_, and **2c**(H_2_O)_2_ are shown in Figure 1. It should be noted from Table 1 that the release of multiple water molecules in processes such as (5a)–(5c) endows a large entropic component favoring complex formation. In addition, release of a proton into a solution buffered to pH = 7 provides an additional 40 kJ mol^−1^ (=*RT* ln⁡⁡(10^−7^)) as a driving force for the forward direction.

Of the three 1 : 1 complexes, only the most stable, **2a**(H_2_O)_2_, is formed exergonically at physiological pH. This structure has a square pyramidal configuration with three-point coordination of the Asp residue to the cupric ion. The amino and carboxylate groups form adjacent corners of the square, and the carbonyl of the amide occupies the axial position. Two water molecules complete the square base. Structure **2b**(H_2_O)_2_, which is lacking the axial carbonyl oxygen coordination, is less stable than **2a**(H_2_O)_2_ by 32 kJ mol^−1^. The primary reason for the lower stability of **2b**(H_2_O)_2_ is enthalpic. The Cu–O bond strength of the coordinated carbonyl oxygen of Asp1 is about 50 kJ mol^−1^ (Table 1). The most stable structure with a deprotonated amide N coordinated to the Cu(II) is **2c**(H_2_O)_2_, which is 47 kJ mol^−1^ less stable than **2a**(H_2_O)_2_. The missing proton is on the carboxylate group. Its p*K*_*a*_ is predicted to be 13. In **2c**(H_2_O)_2_, the amide N and the N-terminal NH_2_ groups occupy adjacent corners of the square pyramidal base with two waters in the other two corners. The neutral carboxylic acid group of **2c** is attached to the axial position with a long bond (2.43 Å, compared to the average Cu-OH_2_ distance, 2.08 Å) [43]. 

### 4.1. Interaction of Cu^**2**+^(aq) with the N-Terminal Asp1Ala2 **3**

The amide-deprotonated structure, **2c**(H_2_O)_2_, is not predicted to be stable in water. However, such a structure permits additional chelation by the carbonyl of the same residue, Ala2 in the present model system. Thus, **3** (i.e., Asp1Ala2) (Figure 2) may provide three ligands for Cu^2+^(aq) if the amide group is deprotonated. The bicyclic structure, **4**(H_2_O) (Figure 2), is the most stable structure that has coordination by the N-terminal NH_2_ of Asp1 and the deprotonated amide N and carbonyl O of Ala2 to the Cu^2+^. All three groups occupy sites in the equatorial coordination plane, the last site being occupied by a water molecule. A second water H bonds to the first and the carboxylic acid group rather than to the copper. The carboxylate group of Asp1 is protonated and interacting with the copper only through H-bonding to the bound water. Equation (6) examines the stability of **4**(H_2_O) relative to the dissociated species:

(6) $$\begin{array}{cc} 3+\text{Cu}{\left({\text{H}}_{2}\text{O}\right)}_{5}\to 4\left({\text{H}}_{2}\text{O}\right)+4{\text{H}}_{2}\text{O}+{\text{H}}^{+}; & \\ \Delta G_{\left(6\right)}=10.2\;{\text{kJ}\;\text{mol}}^{-1}, & \end{array}$$

where Δ*G*_(6)_ has been adjusted to pH = 7. Thus, compared to (5c), coordination by the O of Ala2 provides an additional 19 kJ mol^−1^ of stabilization but is not enough to render the complex stable in water at physiological pH. The predicted p*K*_*a*_ of the carboxylate proton of **4**(H_2_O) is 10, indicating that the deprotonated form of **4**(H_2_O) *would* be stable at pH = 9.

### 4.2. Interaction of Cu^**2**+^/Asp1 Complex, **2a**(H_2_O)_2_, with Imidazole (**Im**)

The side chain of His6 is modeled by imidazole (**Im**). Interaction of Cu^2+^(aq) with one and two His is represented by:

(7a) $$\begin{array}{cc} Im+\text{Cu}{{\left({\text{H}}_{2}\text{O}\right)}_{5}}^{2+}\to \text{Cu}\left(Im\right){{\left({\text{H}}_{2}\text{O}\right)}_{4}}^{2+}+{\text{H}}_{2}\text{O}; & \\ \Delta G_{\left(7\text{a}\right)}=-0.5\;{\text{kJ}\;\text{mol}}^{-1}, & \end{array}$$

(7b) $$\begin{array}{cc} 2Im+\text{Cu}{{\left({\text{H}}_{2}\text{O}\right)}_{5}}^{2+}\to \text{Cu}{\left(Im\right)}_{2}{{\left({\text{H}}_{2}\text{O}\right)}_{3}}^{2+}+2{\text{H}}_{2}\text{O}; & \\ \Delta G_{\left(7\text{b}\right)}=-10.9\;{\text{kJ}\;\text{mol}}^{-1}. & \end{array}$$

The structures of the two **Im**/Cu(II) complexes are shown in Figure 1. Displacement of a water ligand by **Im** is predicted to be isoergonic, Δ*G*_(7b)_ = −0.5 kJ mol^−1^. This result derives from the near cancellation of a highly exergonic change in the gaseous phase by a correspondingly large loss of free energy of solvation (the Δ*G*_(g)_ and ΔΔ*G*_solv_ terms in Table 1). It is in disagreement with results derived from early pH measurements which found log_10_ *β*_1_ = 3.76 or Δ*G*_1_ = −21 kJ mol^−1^ [44]. Addition of the second **Im** was found experimentally to have log_10_ *β*_2_ = 3.39 or Δ*G*_1_ = −19 kJ mol^−1^ [44]. The displacement of a second water is calculated to be more favored, by 10 kJ mol^−1^. The computed results suggest that a monoadduct will disproportionate to form the bisadduct and aqueous Cu(II). The preferred orientation of the imidazole rings in Cu(**Im**)_2_(H_2_O)_3_ is perpendicular to the basal square plane. In the bis(**Im**) complex, both *cis*- and *trans*-diastereomers are stable and are nearly isoergonic. In the *cis*-structure, only one of the imidazole rings is almost perpendicular to the basal plane. The single **Im** in Cu(**Im**)  (H_2_O)_4_^2+^ is intended to represent His6 or one of His13 or His14. The second **Im** of Cu(**Im**)_2_(H_2_O)_3_^2+^ may be one of His13 or His14, or a His6 from a second A*β*. Thus, the 1 : 1 interaction of Cu^2+^(aq) with **Im** is less favorable than with Asp1 by 18 kJ mol^−1^ and the 1 : 2 interaction is comparable. 

The most stable structures, **2a**(**Im**)(H_2_O) and **2a**(**Im**)_2_, for the 1 : 1 : 1 and 1 : 1 : 2 complexes, respectively, between Cu^2+^(aq), Asp1, and **Im** are shown in Figure 1. Structure **2a**(**Im**)(H_2_O) is related to **2a**(H_2_O)_2_ by the substitution of the water that is anti to the carboxylate group by **Im**. The basal plane with the axially coordinated amide carbonyl is preserved. Substitution in **2a**(H_2_O)_2_ of the water anti to the amino group yielding **2b**(**Im**)(H_2_O) results in loss of the axial ligand and distortion of the basal plane. Structure **2a**(**Im**)(H_2_O) is more stable than **2b**(**Im**)(H_2_O) by 38 kJ mol^−1^ (from data in Table S2). 

Substitution of both waters of **2a**(H_2_O)_2_ by **Im** yields **2a**(**Im**)_2_. A second structure, **2b**(**Im**)_2_, in which the **Im** residues are opposite the NH_2_ and amide carbonyl groups, and the carboxylate group occupies the axial position, is also stable. In **2b**(**Im**)_2_, the carboxylate group is coordinated through one of the oxygen atoms but perpendicular to the CO_2_ plane. Structure **2a**(**Im**)_2_ is more stable than **2b**(**Im**)_2_ (Figure 1) by 11 kJ mol^−1^. Equations (8a) and (8b) explore the possible reactions that may yield the 1 : 1 : 1 Cu^2+^(aq), Asp1, and **Im** adduct, **2a**(**Im**)(H_2_O):

(8a) $$\begin{array}{cc} \begin{array}{c} 2a{\left({\text{H}}_{2}\text{O}\right)}_{2}+Im\to 2a\left(Im\right)\left({\text{H}}_{2}\text{O}\right)+{\text{H}}_{2}\text{O}; \end{array} & \\ \Delta G_{\left(8\text{a}\right)}=-24.1\;\text{kJ}\;{\text{mol}}^{-1}, & \end{array}$$

(8b) $$\begin{array}{cc} 1+\text{Cu}\left(Im\right){{\left({\text{H}}_{2}\text{O}\right)}_{4}}^{2+}\to 2a\left(Im\right)\left({\text{H}}_{2}\text{O}\right)+3{\text{H}}_{2}\text{O}+{\text{H}}^{+}; & \\ \Delta G_{\left(8\text{b}\right)}=-41.6\;\text{kJ}\;{\text{mol}}^{-1}, & \end{array}$$

where Δ*G*_(8b)_ has been adjusted to pH = 7. Thus, **2a**(**Im**)(H_2_O) is stable with respect to dissociation either by releasing an imidazole ligand or the N-terminal Asp. In the context to A*β*, these results imply that Asp1 **1** and any of the His residues may form a stable complex with Cu(II) in water at physiological pH. 

The possible formation of the 1 : 1 : 2 complex, **2a**(**Im**)_2_, is explored in reactions (9a) and (9b):

(9a) $$\begin{array}{cc} 2a\left(Im\right)\left({\text{H}}_{2}\text{O}\right)+Im\to 2a{\left(Im\right)}_{2}+{\text{H}}_{2}\text{O}; & \\ \Delta G_{\left(9\text{a}\right)}=-11.0{\;\text{kJ}\;\text{mol}}^{-1}, & \end{array}$$

(9b) $$\begin{array}{cc} 1+\text{Cu}{\left(Im\right)}_{2}{{\left({\text{H}}_{2}\text{O}\right)}_{3}}^{2+}\to 2a{\left(Im\right)}_{2}+3{\text{H}}_{2}\text{O}+\;{\text{H}}^{+}; & \\ \Delta G_{\left(9\text{b}\right)}=-42.2\;\text{kJ}\;{\text{mol}}^{-1}, & \end{array}$$

where Δ*G*_(9b)_ has been adjusted to pH = 7. Thus, as with **2a**(**Im**)(H_2_O), the 1 : 1 : 2 complex, **2a**(**Im**)_2_, is also stable toward dissociation, and, in the context to A*β*, these results imply that Asp1, His6, and either of His13 or His14 may form stable complexes in water at physiological pH. 

By (6), it was apparent that deprotonation of the amide N (of Ala2) required coordination of the O of Ala2 in order to afford a complex, **4**(H_2_O), that could be formed at pH = 9 but was not stable at pH = 7. Equations (10a) and (10b) examine the possibility that the water of **4**(H_2_O) may be displaced by **Im**:

(10a) $$\begin{array}{cc} 4\left({\text{H}}_{2}\text{O}\right)+Im\to 4\left(Im\right)+{\text{H}}_{2}\text{O}; & \\ \Delta G_{\left(10\text{a}\right)}=-20.5\;\text{kJ}\;{\text{mol}}^{-1}, & \end{array}$$

(10b) $$\begin{array}{cc} 3+\text{Cu}{{\left({\text{H}}_{2}\text{O}\right)}_{5}}^{2+}+Im\to 4\left(Im\right)+5{\text{H}}_{2}\text{O}+{\text{H}}^{+}; & \\ \Delta G_{\left(10\text{b}\right)}=-10.3\;\text{kJ}\;{\text{mol}}^{-1}, & \end{array}$$

where Δ*G*_(10b)_ has been adjusted to pH = 7. Thus, the additional stabilization afforded by replacing water by **Im** (10a), is sufficient to render the product, **4**(**Im**) (Figure 2), stable in water at physiological pH (10b), but would have only marginal stability at lower pH, Δ*G*_(10b)_ = −4 kJ mol^−1^ at pH = 6. Structure **4**(**Im**) has a tetracoordinate square planar configuration. The carboxylate group of Asp1 is H-bonded to the NH_2_ group and not interacting with the copper. Deprotonation of **4**(**Im**) affords **4**(**Im**)(CO_2_^−^) (Figure 2) with p*K*_*a*_ = 11. **4**(**Im**)(CO_2_^−^) is pentacoordinated with the carboxylate group occupying the fifth site. In the context of A*β*, (10a) and (10b) imply that a 3N1O complex incorporating the N-terminal NH_2_ group, the carbonyl O, and the deprotonated amide N of Ala2, and one of His6, His13 or H14, should be observed at physiological pH:

(11) $$\begin{array}{cc} 4\left(Im\right)+Im\to 4{\left(Im\right)}_{2}; & \\ \Delta G_{\left(11\right)}=6.9\;{\text{kJ}\;\text{mol}}^{-1}. & \end{array}$$

By (11), coordination of a second **Im** to the vacant axial coordination site of **4**(**Im**) to yield **4**(**Im**)_2_ (Figure 2) is unfavorable by 7 kJ mol^−1^. Such a structure may be an intermediate for the interchange of the His residues of A*β*. 

But is a deprotonated amide structure like **4**(**Im**) stable compared to a form like **2a**(**Im**)_2_ in which the amide is protonated and Ala2 is not involved? Equation (12) compares the stability of the most stable copper-coordinated structure that uses both Asp1 and Ala2, **4**(**Im**), with one that does not involve Ala2, namely, **2a**(**Im**)_2_:

(12) $$\begin{array}{cc} 4\left(Im\right)+Im+1\to 2a{\left(Im\right)}_{2}+3; & \\ \Delta G_{\left(12\right)}=-42.8\;{\text{kJ}\;\text{mol}}^{-1}. & \end{array}$$

The relatively large exergonicity of (12), Δ*G*_(12)_ = −43 kJ mol^−1^, strongly suggests that Ala2 is *not* involved in the bonding in the N-terminal copper-bound species. The principal reason, from Table 1, is enthalpic (Δ*H*_(12)_ = −84 kJ mol^−1^ and is a consequence of the greater acidity of the carboxylate group than of the amide group. Thus, **2a**(**Im**)_2_ is the closest model for Component I of A*β*, but raising the pH, at least in the physiological range, does not lead to Component II as proposed by Faller and Hureau and coworkers [6, 7]. In the following section, we propose another structure for Component II and discuss the nature of the pH dependence.

### 4.3. Interaction of Cu^2+^ with His13His14 **5**

We explore below the special case of His13His14 where the **Im** groups of the two His residues are tethered by an intervening amide link. The tethering has an important consequence. As with **3**, this configuration permits three-point chelation to the copper, a favorable contribution to the free energy of binding, but without the penalty of amide deprotonation. 

The His13His14 sequence of A*β* is modeled by **5 **(Figure 3), in which only the two side chains and the intervening amide link are preserved [17]. The interaction between **5** and Cu^2+^(aq) yielding **6**(H_2_O)_2_ was recently studied experimentally and reexamined theoretically by the procedures employed in the present paper [18]. The calculated association constant, log⁡_10_⁡*K*_(12)_ for (13), was in good agreement with the experimental value, log_10_ *K*_as_ = 5.6 [18]:

(13) $$\begin{array}{cc} 5+\text{Cu}{{\left({\text{H}}_{2}\text{O}\right)}_{5}}^{2+}\to \;6{\left({\text{H}}_{2}\text{O}\right)}_{2}+3{\text{H}}_{2}\text{O}; & \\ \Delta G_{\left(13\right)}=-36.0\;\text{kJ}\;{\text{mol}}^{-1};\;\log_{10}K_{\left(13\right)}=6.3. & \end{array}$$

Structure **6**(H_2_O)_2_, which models the mode of attachment of Cu(II) to His13His14, has the two **Im** groups in the *trans*-positions of a distorted square plane. The backbone amide carbonyl oxygen and a water molecule occupy the other two opposing sites. A second water occupies the apical site of the square pyramid. The *trans*-orientation of the two **Im** groups is the favored mode of attachment as seen in *trans*-Cu(**Im**)_2_(H_2_O)_3_ (Figure 1). It has been argued that the copper does not attach to a HisHis sequence through the N_*δ*_ atoms in a *trans*-arrangement as in **6**(H_2_O)_2_, but rather through the N_*ε*_ atoms in a *cis*-arrangement [1]. The latter is the configuration seen in the crystal structure of bis(cyclo-L-histidyl-L-histidyl)copper(II), the cyclic anhydride of histidine [45]. In this compound, Cu^2+^ chelates to the N_*ε*_ atom of the imidazole rings in a *cis*-arrangement. In the present system, the most stable structure in which Cu(II) is attached to **5** with the cis coordination pattern, is 6^**εε**^(H_2_O)_3_ (Tables S1 and S2)). Structure 6^**εε**^(H_2_O)_3_ is predicted to be less stable relative to **6**(H_2_O)_2_ + H_2_O by 36 kJ mol^−1^. The constraint imposed by the framework of the cyclic anhydride of histidine does not permit bridging of the copper ion through one or both N_*δ*_ atoms of the **Im** groups. In **5**, or in monomeric A*β*, there are no such constraints. We also found structures, 6^**δε**^(H_2_O)_2_ and 6^**εδ**^(H_2_O)_2_ (Tables S1 and S2)), in which coordination is through one N_*δ*_ and one N_*ε*_ nitrogen of **5**. These also have the cis orientation and are even less stable, 46 and 51 kJ mol^−1^ (data in Table S2). We do not consider these structures to be relevant to the chemistry of Cu/A*β* in Alzheimer's disease and do not discuss them further.  

Species **6**(H_2_O)_2_ may add an additional **Im** residue yielding, **6**(**Im**)(H_2_O). The results are presented in:

(14) $$\begin{array}{cc} 6{\left({\text{H}}_{2}\text{O}\right)}_{2}+Im\to 6\left(Im\right)\left({\text{H}}_{2}\text{O}\right)+{\text{H}}_{2}\text{O}\;; & \\ \Delta G_{\left(14\right)}=-12.5\;\text{kJ}\;{\text{mol}}^{-1}. & \end{array}$$

The remaining water is in an axial orientation with a long Cu-O separation, 2.40 Å. Exchange of the water by an O of an amide carbonyl group (of NMA = N-methylacetamide) yields **7** (Figure 3). In **7**, the two carbonyl groups occupy equivalent positions in the equatorial plane of a trigonal bipyramidal configuration about the Cu(II) ion. Attempts to optimize square planar structures with the O of His13 or NMA in an axial position converged to similar trigonal bipyramidal structures. The reaction is described in:

(15) $$\begin{array}{cc} 6\left(Im\right)\left({\text{H}}_{2}\text{O}\right)+\text{NMA}\to 7+{\text{H}}_{2}\text{O}; & \\ \Delta G_{\left(15\right)}=-20.5\;\text{kJ}\;{\text{mol}}^{-1};\;\log_{10}K_{\left(15\right)}=3.6. & \end{array}$$

Reaction (15) is moderately exergonic in water, indicating that the Cu(II) environment could consist of the three His residues and an additional carbonyl group. In the context of Cu/A*β*, the experimental results of Barnham and coworkers [12], and the theoretical modeling of Sodupe and coworkers [14], on Cu(II)/A*β*(1-16), the obvious candidate for the additional carbonyl O is Ala2. We note however that displacement of water by NMA is strongly *endothermic* in the gaseous phase, Δ*H*_(g)_ = 68 kJ mol^−1^ (Table 1). This is a consequence of the steric crowding about the Cu(II) site that forces the unusual trigonal bipyramidal geometry. The exergonicity of reaction (15) in water ensues from an even larger favorable change in the free energy of solvation, ΔΔ*G*_(aq)_ = −84 kJ mol^−1^ (Table 1). Solvation was also found to favor the coordination of the O of Ala2 in the work of Sodupe and coworkers [14].

### 4.4. Interaction of Cu^**2**+^/Asp1Complex, **2a**(H_2_O), with His13His14 **5**

The most stable structures corresponding to the 1 : 1 : 1 complex between Cu^2+^(aq), Asp1, and His13His14 (**5**), namely, **8a **and **8b**, are shown in Figure 2. The more stable of the two, **8a**, has a square pyramidal coordination sphere about the copper, with bidentate coordination of the –NH_2_ and –CO_2_^−^ groups of the Asp in the basal square. One of the **Im** groups (formally of His13) and the backbone amide carbonyl group of **5** form the other two corners. The other **Im **group (formally of His14) has moved into the apical site. The second structure, **8b**, which is less stable by 18 kJ mol^−1^, has the same square pyramidal 3N1O basal configuration as **8a,** but the apical **Im** group has released. Possible routes for the formation of the more stable isomer **8a** are examined in:

(16a) $$\begin{array}{cc} 1+6{\left({\text{H}}_{2}\text{O}\right)}_{2}\to 8a+2{\text{H}}_{2}\text{O}+{\text{H}}^{+}; & \\ \Delta G_{\left(16\text{a}\right)}=+2.8\;\text{kJ}\;{\text{mol}}^{-1}, & \end{array}$$

(16b) $$\begin{array}{cc} 2a{\left({\text{H}}_{2}\text{O}\right)}_{2}+5\;\to 8a+2{\text{H}}_{2}\text{O}; & \\ \Delta G_{\left(16\text{b}\right)}=-15.2\;\text{kJ}\;{\text{mol}}^{-1}, & \end{array}$$

where Δ*G*_(16a)_ has been adjusted to pH = 7. The negative free energy change of (16b), Δ*G*_(16b)_ = −15 kJ mol^−1^, indicates that if the cupric ion was already attached to **2a**(H_2_O)_2_, that is, the N-terminus, it can also associate with **5**, that is, His13His14. However, the small positive value, Δ*G*_(16a)_ = +3 kJ mol^−1^, indicates that **8a** would be partially dissociated, releasing the N-terminal Asp. Equation (17a) addresses the question of whether the N-terminus can be coordinated if there are already three His residues coordinated to the copper ion. Such a structure, **9**, is shown in Figure 3:

(17a) $$\begin{array}{cc} 1+6\left(Im\right)\left({\text{H}}_{2}\text{O}\right)\to 9+{\text{H}}_{2}\text{O}+{\text{H}}^{+}; & \\ \Delta G_{\left(17\text{a}\right)}=+22.0\;{\text{kJ}\;\text{mol}}^{-1}, & \end{array}$$

(17b) $$\begin{array}{cc} 9\to 8a+Im; & \\ \Delta G_{\left(17\text{b}\right)}=-6.7\;{\text{kJ}\;\text{mol}}^{-1}, & \end{array}$$

(17c) $$\begin{array}{cc} 9+{\text{H}}_{2}\text{O}+{\text{H}}^{+}\to 5+2a\left(Im\right)\left({\text{H}}_{2}\text{O}\right); & \\ \Delta G_{\left(17\text{c}\right)}=24.4\;\text{kJ}\;{\text{mol}}^{-1}, & \end{array}$$

where Δ*G*_(17a)_ and Δ*G*_(17c)_ have been adjusted to pH = 7. The moderately high value, Δ*G*_(17a)_ = 22 kJ mol^−1^, indicates that simultaneous attachment of all four potential ligands to Cu(II), the N-terminal Asp and the three histidines, is not likely at physiological pH. Equations (17b) and (17c) indicate that such an arrangement would be unstable with respect to loss of one histidine but not two. 

In the context of A*β*, the overall picture that emerges from the above considerations is that the N-terminus and all three His residues may not be simultaneously associated with the cupric ion. However, such a structure could be a transitional form connecting more stable structures in which the Cu^2+^(aq) is attached to His6, His13, and His14 (**6**(**Im**)(H_2_O) or **7**) (Figure 3) or to the N-terminus and *two* of the three His residues, His6 and His13, or His6 and His14 (both modeled by **2a**(**Im**)_2_) (Figure 1) but not His13 and His14 (**8a** is unstable by (16a)) (Figure 3). Thus, structure **2a**(**Im**)_2_, with 3N1O equatorial coordination to Cu(II), serves as a model for Components Ia and Ib. In each case the equatorial O ligand is one of the carboxylate O atoms of Asp1. Structures **6**(**Im**)(H_2_O) or **7** serve as models for Component II. **6**(**Im**)(H_2_O) has the observed 3N1O coordination pattern. Structure **7** is preferred because it is more stable, but it formally has a 3N2O coordination pattern in a trigonal bipyramid. The computed results for Cu(II) species agree in most respects with experimental expectations, except possibly for the nature of the O ligand, which would be the carbonyl O atom of His13 if **6**(**Im**)(H_2_O) proves to be the better model for Component II. However, favoring **7** in this respect also is the experimental [12] and other computational [14] evidence that the “O” should be the O atom of Ala2 provided the approximately equivalent equatorial O atoms of the trigonal bipyramidal geometry would manifest as 3N1O coordination in EPR experiments. Attempts on our part to completely displace the O of His13 in Cu(II) complexes by any other ligand always failed. 

If Component I is modeled by **2a**(**Im**)_2_ and Component II is modeled by **7**, what then is the nature of the pH dependence that shifts the equilibrium from one to the other in the narrow physiological pH range? We suggest that, since the p*K*_*a*_ of His residues is in this range, one or more of the His residues would be protonated. The presence of the Cu^2+^ ion sets up a delicate balance: in Component I, either His13 or His14 is protonated, permitting the other to bind to the copper together with His6 and Asp1 (but not Ala2); at a slightly higher pH, the remaining His is deprotonated and all three can bind to the copper, displacing Asp1 but leaving the nearby O of Ala2 attached.

## 5. Interaction of Cu^+^(aq) with the N-Terminal Asp1 **1**

**Figure 4:**
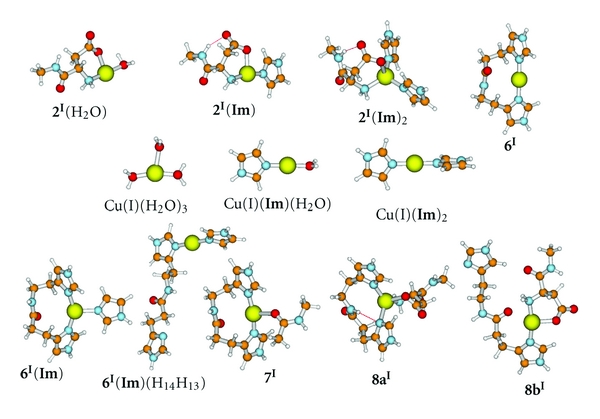
Stable structures with Cu(I). Ball colors: Cu = large yellow; C = orange; N = blue; O = red; H = white.

The most stable form of the aqueous cuprous ion at the present theoretical level is the T-shaped triaqua structure, Cu(H_2_O)_3_^+^ (Figure 4), Reaction with Asp1 **1** yielded several aquated structures with different patterns of chelation. The most stable of these is the 1 : 1 Cu(I) : Asp1 complex, 2^**I**^(H_2_O) (Figure 4, see the following equation):

(18) $$\begin{array}{cc} 1+{\text{Cu}{\left({\text{H}}_{2}\text{O}\right)}_{3}}^{+}\to \;2^{I}\left({\text{H}}_{2}\text{O}\right)+2{\text{H}}_{2}\text{O}+{\text{H}}^{+}; & \\ \Delta G_{\left(18\right)}=+5.3\;\text{kJ}\;{\text{mol}}^{-1}, & \end{array}$$

where Δ*G*_(18)_ has been adjusted to pH = 7. We denote structures containing the cuprous ion by the superscript **I** on the structure number of the Cu(II) equivalent. As with Cu(H_2_O)_5_^2+^ and Cu(H_2_O)_3_^+^, the cuprous form of a complex will generally have fewer ligands than the equivalent cupric form. Some or all of the attached H_2_O will be lost upon reduction of the copper. If there is no attached water, then one or more of the coordinated ligands will be released upon reduction. The structures of all complexes containing Cu(I) are shown in Figure 4.

The small endergonic free energy change for reaction (18) in water, Δ*G*_(18)_ = 5 kJ mol^−1^ at pH = 7, suggests that there is a small amount of 1 : 1 complex formed between Cu^+^(aq) and Asp1 under physiological conditions. We examine whether complexation of Asp1 is feasible if the cuprous ion is already attached to one or more imidazoles. The reactions for the 1 : 1 complex between Cu^+^(aq) and **Im**, Cu(**Im**)(H_2_O)^+^, is given in:

(19) $$\begin{array}{cc} Im+{\text{Cu}{\left({\text{H}}_{2}\text{O}\right)}_{3}}^{+}\to \text{Cu}\left(Im\right){\left({\text{H}}_{2}\text{O}\right)}^{+}+2{\text{H}}_{2}\text{O}; & \\ \Delta G_{\left(19\right)}=-16.3\;\text{kJ}\;{\text{mol}}^{-1}. & \end{array}$$

Thus, Cu(**Im**)(H_2_O)^+^ is stable in water and the free energy of complexation is higher than for the equivalent Cu(II) complex (7a). The following reactions explore the addition of a second imidazole group to Cu(**Im**)(H_2_O)^+^, either as free **Im** or as His13 or His14 of **5**, yielding products Cu(**I** **m**)_2_^+^ (20a), Cu(**Im**)(H_13_H_14_)^+^ (20b), and Cu(**Im**)(H_14_H_13_)^+^ (20c), respectively:

(20a) $$\begin{array}{cc} Im+\text{Cu}\left(Im\right){\left({\text{H}}_{2}\text{O}\right)}^{+}\to {\text{Cu}{\left(Im\right)}_{2}}^{+}+{\text{H}}_{2}\text{O}; & \\ \Delta G_{\left(20\text{a}\right)}=-27.4\;\text{kJ}\;{\text{mol}}^{-1}, & \end{array}$$

(20b) $$\begin{array}{cc} 5+\text{Cu}\left(Im\right){\left({\text{H}}_{2}\text{O}\right)}^{+}\to \text{Cu}\left(Im\right){\left({\text{H}}_{13}{\text{H}}_{14}\right)}^{+}+{\text{H}}_{2}\text{O}; & \\ \Delta G_{\left(20\text{b}\right)}=-43.4\;\text{kJ}\;{\text{mol}}^{-1}, & \end{array}$$

(20c) $$\begin{array}{cc} 5+\text{Cu}\left(Im\right){\left({\text{H}}_{2}\text{O}\right)}^{+}\to \text{Cu}\left(Im\right){\left({\text{H}}_{14}{\text{H}}_{13}\right)}^{+}+{\text{H}}_{2}\text{O}; & \\ \Delta G_{\left(20\text{c}\right)}=-31.1\;\text{kJ}\;{\text{mol}}^{-1}. & \end{array}$$

Thus, addition of His14 of** 5** is approximately equivalent energetically to the addition of a free **Im** group and both are more exergonic than addition of a single **Im** (19). Addition of His13 (20b) is more exergonic still due to a higher free energy of solvation (Table 1) which ensues as a consequence of the higher dipole moment of Cu(**Im**)(H_13_H_14_)^+^, *μ* = 15.4 D compared to *μ* = 13.2 D for Cu(**Im**)(H_14_H_13_)^+^.

Both 1 : 1 : 1 and 1 : 1 : 2 complexes between Cu(I), Asp1 **1**, and **Im**, namely, 2^**I**^(**I** **m**) and 2^**I**^(**Im**)_2_ (Figure 4), are stable in the gaseous phase. Formation of the 1 : 1 : 1 complex, 2^**I**^(**Im**) (Figure 4), is essentially isoergonic at pH = 7 if one **Im** is already attached to Cu(I):

(21) $$\begin{array}{cc} 1+\text{Cu}\left(Im\right){\left({\text{H}}_{2}\text{O}\right)}^{+}\to 2^{I}\left(Im\right)+{\text{H}}_{2}\text{O}+{\text{H}}^{+}; & \\ \Delta G_{\left(21\right)}=-3.0\;\text{kJ}\;{\text{mol}}^{-1}, & \end{array}$$

where Δ*G*_(21)_ has been adjusted to pH = 7. Addition of a second **Im** to form the 1 : 1 : 2 complex, 2^**I**^(**Im**)_2_ (Figure 4), is endergonic, Δ*G*_(22)_ = 23 kJ mol^−1^:

(22) $$\begin{array}{cc} 2^{I}\left(Im\right)+Im\to 2^{I}{\left(Im\right)}_{2}; & \\ \Delta G_{\left(22\right)}=22.7\;\text{kJ}\;{\text{mol}}^{-1}. & \end{array}$$

Because of the high affinity of Cu(I) for two imidazoles, addition of Asp1 **1** to the bis(imidazole)Cu(I) complex will not occur:

(23) $$\begin{array}{cc} 1+\text{Cu}{\left(Im\right)}_{2}^{+}\to 2^{I}{\left(Im\right)}_{2}+{\text{H}}^{+}; & \\ \Delta G_{\left(23\right)}=47.2\;\text{kJ}\;{\text{mol}}^{-1}, & \end{array}$$

where Δ*G*_(23)_ has been adjusted to pH = 7. On the basis of this result, we have not attempted to add Asp1 to Cu(**Im**)(H_13_H_14_)^+^ or Cu(**Im**)(H_14_H_12_)^+^. 

### 5.1. Interaction of Cu^+^ and/Asp1 **1**, with His13His14 **5**

Cu(I) binds to the HisHis region **5** yielding the 1 : 1 complex, 6^**I**^ (Figure 4), with high affinity,

(24) $$\begin{array}{cc} 5+{\text{Cu}{\left({\text{H}}_{2}\text{O}\right)}_{3}}^{+}\to 6^{I}+3{\text{H}}_{2}\text{O}; & \\ \Delta G_{\left(24\right)}=-72.8\;\text{kJ}\;{\text{mol}}^{-1};\;\log_{10}K_{\left(24\right)}=12.7. & \end{array}$$

Coordination of additional waters to 6^**I**^ is endergonic, for example, Δ*G* ≥ 15 kJ mol^−1^ for the addition of one water (data not shown). The calculated affinity constant, log_10_ *K*_(23)_ = 12.7, is in very good agreement with that found for the Cu(I)/A*β*(1-16) complex, log_10_ *K*_*aff*⁡_ ≈ 14 [24]. The value is substantially higher than for the addition of two free imidazoles to Cu^+^(aq) (20a), or a free **Im** and one of the two imidazoles of **5** ((20b) and (20c)), thus highlighting the importance of chelation. It is also higher than for the addition of Cu^2+^(aq) to HisHis (13). The calculations clearly confirm [30] that Cu(I) will bind more strongly than Cu(II) to A*β* and that the preferred site of binding of Cu(I) is His13His14. The linear geometry and a Cu-N distance of 1.877 Å in Cu(I)/A*β*(1-40) was deduced from fitted EXAFS data by Shearer and Szalai [23]. Our calculated value for 6^**I**^, 1.894 Å, is in good agreement. 

Addition of Asp1 **1** to the linear Cu(I)/HisHis complex, 6^**I**^, in the gaseous phase yields two stable structures, 8**a** ^**I**^ and 8**b** ^**I**^ (Figure 4), of equal energy in water. However, the formation of either by addition of Asp1 **1** to 6^**I**^ in water is highly endergonic ((25a) and (25b)) due to the high stability of 6^**I**^:

(25a) $$\begin{array}{c} 1+6^{I}\to 8a^{I}\;+{\text{H}}^{+};\;\Delta G_{\left(25\text{a}\right)}=64.6\;\text{kJ}\;{\text{mol}}^{-1}, \end{array}$$

(25b) $$\begin{array}{c} 1+6^{I}\to 8b^{I}\;+{\text{H}}^{+};\;\Delta G_{\left(25\text{b}\right)}=63.4\;\text{kJ}\;{\text{mol}}^{-1}, \end{array}$$

where Δ*G*_(25a)_ and Δ*G*_(25b)_ have been adjusted to pH = 7. Equations (16a), (16b), and (17a) indicate that the N-terminus will be weakly associated with Cu(II) complexes of two of the His residues. However, upon reduction of the copper to Cu(I), the N-terminus will be released leaving only the linear Cu(I)/HisHis structure. Equation (15) indicates that an amide carbonyl will be weakly associated with Cu(II) complexes of all three His residues. Equation (26) examines whether the carbonyl would remain attached to the reduced copper species, 6^**I**^:

(26) $$\begin{array}{cc} \text{NMA}+6^{I}\to 7^{I}; & \\ \Delta G_{\left(26\right)}=25.0\;\text{kJ}\;{\text{mol}}^{-1}. & \end{array}$$

The complex with NMA (N-methylacetamide), 7^**I**^ (Figure 3), is strongly bound in the gaseous phase, Δ*H*_((26),g)_ = −59 kJ mol^−1^, but is formed endergonically in water due to a combination of loss of solvation free energy and an unfavorable entropic term (Table 1). Addition of **Im** to 6^**I**^ to yield 6^**I**^(**Im**) (Figure 4) is slightly endergonic,

(27) $$\begin{array}{cc} Im+6^{I}\to 6^{I}\left(Im\right); & \\ \Delta G_{\left(27\right)}=10.1\;\text{kJ}\;\text{mol}-1;\;\log_{10}K_{\left(27\right)}=-1.8. & \end{array}$$

As with NMA, the addition reaction is endergonic principally by virtue of loss of free energy of solvation, but also in part due to the unfavorable change in entropy (Table 1).

In the context of a reduced copper/A*β* complex, there is ample experimental evidence that Cu(I) is bound to His13His14 precisely as depicted in 6^**I**^, in a linear fashion through the N_*δ*_ of both **Im** groups (Figure 4) [21, 22, 46]. All other ligands, including the third **Im**, are released upon reduction. The **Im** (His6) is tethered to the Cu(I) binding region at His13His14, and the loss of entropy may be less, reducing the endergonicity of (27). There is experimental evidence that a third **Im** can associate transiently with Cu(I)/A*β* [26].

## 6. Reduction Potentials for Cu(II) Complexes with the N-Terminal Asp1 **1**

Computed reduction potentials for various combinations of Cu(II)/A*β* and Cu(I)/A*β* coordination patterns, when compared with the experimental value for Cu/A*β*, *E*° ≈ 0.30 V–0.34 V, [28, 29, 47] may serve to distinguish among the different possibilities that have been suggested in the literature and discussed in the Introduction. Since all experimental indications suggest that A*β* and Cu/A*β* are highly fluxional species, the most stable structures should be most populated. We assume that electrochemical reduction is an equilibrium process. Logically then, reduction of the (predicted) most stable Cu(II) species, yielding the most stable Cu(I) species, should yield the most representative value of *E*° or *E*°′. As a second point of reference, the experimental value for the reduction of aqueous cupric ion is *E*°(Cu^2+^(aq)/Cu^+^(aq)) = 0.17 V [41]. We now examine possible redox scenarios in the Cu/A*β* context.

Equation (28) describes the reduction process if the copper was attached only to the N-terminal Asp1:

(28) $$\begin{array}{cc} 2a{\left({\text{H}}_{2}\text{O}\right)}_{2}+{\text{e}}^{-}\to 2a^{I}\left({\text{H}}_{2}\text{O}\right)+{\text{H}}_{2}\text{O}; & \\ E^{{}^{\circ}}\left(2a{\left({\text{H}}_{2}\text{O}\right)}_{2}/2a^{I}\left({\text{H}}_{2}\text{O}\right)\right)=-0.07\;\text{V}. & \end{array}$$

The calculated standard reduction potential *E*°(**2a**(H_2_O)_2_/2**a** ^**I**^(H_2_O)) = −0.07 V, is lower than the value for aqueous copper. Therefore, Cu(II) cannot be attached only to the N-terminus since the lower *E*° is incompatible with the experimental observation that the reduction potential is elevated. A lower value is expected since a negatively charged group is attached to the Cu(II), thereby lowering the net charge of the oxidized species.

Equation (29) describes reduction of copper attached in a 1 : 1 ratio to Asp1 and **Im** The Cu(II) species, **2a**(**Im**)(H_2_O), was found to be stable in water ((8a) and (8b)), but the Cu(I) species, 2^**I**^(**I** **m**), had only transient stability (21):

(29) $$\begin{array}{cc} 2a\left(Im\right)\left({\text{H}}_{2}\text{O}\right)+{\text{e}}^{-}\to 2^{I}\left(Im\right)+{\text{H}}_{2}\text{O}; & \\ E^{{}^{\circ}}\left(2a\left(Im\right)\left({\text{H}}_{2}\text{O}\right)/2^{I}\left(Im\right)\right)=-0.07\;\text{V}. & \end{array}$$

The result, *E*°(**2a**(**Im**)(H_2_O)/2^**I**^(**I** **m**))) = −0.07 V, indicates that the presence of the **Im** moiety has little effect on the predicted reduction potential. 

With respect to the possibility that Cu(II) may be coordinated to Asp1 and two of the **Im** moieties, the Cu(II) species, **2a**(**Im**)_2_, was found to be stable at pH = 7 ((9a) and (9b)). Structure **2a**(**Im**)_2_ corresponds to Component I, the low pH species, in which Cu(II) is attached to His6 and either His13 or His14 as well as the N-terminus. The corresponding reduced species, 2^**I**^(**Im**)_2_ was found to release the Asp (23). Equations (30a) and (30b) describe the appropriate reduction process if the Cu(I) of Component I remains attached to the same two His residues:

(30a) $$\begin{array}{cc} 2a{\left(Im\right)}_{2}+5\;+{\text{e}}^{-}+{\text{H}}^{+}\to 1+\text{Cu}\left(Im\right){\left({\text{H}}_{13}{\text{H}}_{14}\right)}^{+}; & \\ E^{{}^{\circ}\prime }\left(2a{\left(Im\right)}_{2},{\text{H}}^{+}/\text{Cu}\left(Im\right){\left({\text{H}}_{13}{\text{H}}_{14}\right)}^{+},1\right)=0.24\;\text{V}, & \end{array}$$

(30b) $$\begin{array}{cc} 2a{\left(Im\right)}_{2}+5\;+\;{\text{e}}^{-}+{\text{H}}^{+}\to 1+\text{Cu}\left(Im\right){\left({\text{H}}_{14}{\text{H}}_{13}\right)}^{+}; & \\ E^{{}^{\circ}\prime }\left(2a{\left(Im\right)}_{2},{\text{H}}^{+}/\text{Cu}\left(Im\right){\left({\text{H}}_{14}{\text{H}}_{13}\right)}^{+},1\right)=0.11\;\text{V}. & \end{array}$$

If, during the reduction process, the most stable Cu(I) product, 6^**I**^, is formed, the process may be modeled by (31) in which His6 is retained or by (32) in which His6 is released:

(31) $$\begin{array}{cc} 2a{\left(Im\right)}_{2}+5+{\text{e}}^{-}+{\text{H}}^{+}\to 1+6^{I}\left(Im\right)+Im; & \\ E^{{}^{\circ}\prime }\left(2a{\left(Im\right)}_{2},5,{\text{H}}^{+}/6^{I}\left(Im\right),1,Im\right)=0.27\;\text{V}, & \end{array}$$

(32) $$\begin{array}{cc} 2a{\left(Im\right)}_{2}+5\;+\;{\text{e}}^{-}+{\text{H}}^{+}\to 1+6^{I}+2Im; & \\ E^{{}^{\circ}\prime }\left(2a{\left(Im\right)}_{2},5,{\text{H}}^{+}/6^{I},1,Im\right)=0.37\;\text{V}. & \end{array}$$

In summary, the reduction potentials of Cu(II) attached to the N-terminus and one or two independent His residues ((30a) and (30b)) in which the Cu(I) remains attached to the same two His residues are predicted to be slightly lower than the experimental value for Cu(II)/A*β* complexes, 0.30 V–0.34 V. However, if the Cu(I) rearranges to include both His13 and His14, with or without loss of His6 ((31) and (32), resp.), *E*° values are predicted to be close to the experimental value. Therefore, it is possible that Component I is the species that is observed to undergo reduction. 

Component II, modeled by **6**(**Im**)(H_2_O) or **7**, does not involve the N-terminal Asp1. Reduction of either species in which Cu(II) is coordinated to His13His4 will yield the Cu(I)/HisHis species, 6^**I**^, with the release of all other coordinating ligands. In the instance that Cu(II) is *only* attached to HisHis except for waters (33), an elevated value of *E*° is obtained:

(33) $$\begin{array}{cc} 6{\left({\text{H}}_{2}\text{O}\right)}_{2}+{\text{e}}^{-}\to 6^{I}+2{\text{H}}_{2}\text{O}; & \\ E^{{}^{\circ}}\left(6{\left({\text{H}}_{2}\text{O}\right)}_{2}/6^{I}\right)=0.55\;\text{V}. & \end{array}$$

The high result confirms that **6**(H_2_O)_2_ is also an incomplete description of the bonding in Cu(II)/A*β*. A more representative species is **6**(**Im**)(H_2_O), which models all three His residues coordinated to A*β* with a coordinated water:

(34) $$\begin{array}{cc} 6\left(Im\right)\left({\text{H}}_{2}\text{O}\right)+{\text{e}}^{-}\to 6^{I}+Im+{\text{H}}_{2}\text{O}; & \\ E^{{}^{\circ}}\left(6\left(Im\right)\left({\text{H}}_{2}\text{O}\right)/6^{I},Im\right)=0.42\;\text{V}. & \end{array}$$

The *E*° value (34) is close to but higher than the experimental range. The species, **6**(**Im**)(H_2_O), has a coordinated water molecule which may be exchanged for another ligand. On the basis of the experimental finding that the O atom of Ala2 is in the Cu(II) coordination sphere of Component II, we consider here structure **7**, in which the water is replaced by an amide carbonyl, using N-methylacetamide (NMA) as a model. The substrate, **7,** is shown in Figure 3. The reduction of **7** is described in:

(35) $$\begin{array}{cc} 7+{\text{e}}^{-}\to 6^{I}+Im+\text{NMA}; & \\ E^{{}^{\circ}}\left(7/6^{I},Im,\text{NMA}\right)=0.63\;\text{V}. & \end{array}$$

The *E*° value (35) is substantially higher than the experimental range. 

Thus, it appears that none of mechanisms that involve reduction and *spontaneous* loss of ligands provides a satisfactory description of the reduction process. Balland et al., have recently carried out a detailed study of the kinetics of the reduction/oxidation of Cu/A*β*(1-16) complexes by cyclic voltametry and homogeneous transfer from osmium complexes [47]. The electron transfer rate was found to be extremely slow and required a preorganization by 22 and 16 kJ mol^−1^ to geometries of the Cu(II) and Cu(I) species, respectively, between which the actual electron transfer takes place. In the present model systems, the preorganization of the oxidized species is to release the bound carbonyl oxygen, that is, the reverse of (15) for which Δ*G*_(15)_ = 20 kJ mol^−1^. The preorganization of the reduced species prior to oxidation is to gain the **Im** residue, that is, (27) for which Δ*G*_(27)_ = 10 kJ mol^−1^. Thus, in the Balland et al. scheme, the actual reduction is described by:

(36) $$\begin{array}{cc} 6\left(Im\right)\left({\text{H}}_{2}\text{O}\right)+{\text{e}}^{-}\to 6^{I}\left(Im\right)+{\text{H}}_{2}\text{O}; & \\ E^{{}^{\circ}}\left(6\left(Im\right)\left({\text{H}}_{2}\text{O}\right)/6^{I}\left(Im\right),{\text{H}}_{2}\text{O}\right)=0.31\;\text{V}. & \end{array}$$

The calculated reduction potential, *E*°(**6**(**Im**)(H_2_O)/6^**I**^(**Im**),H_2_O) = 0.31 V, is in excellent agreement with that measured by Balland et al., *E*° = 0.30 V, and the calculated free energy changes for the preorganization steps are also in good agreement.

## 7. Conclusions

High-level ab initio electronic structure calculations were applied to models of the N-terminus of A*β*, as well as Ala2, His6, and His13His14, to predict structures of the complexes of Cu(II) and Cu(I) in water at physiological pH. The calculated binding affinities of both Cu(II) and Cu(I) to the His13His14 model, log_10_ *K*_*aff*⁡_ = 6.3 (13) and log_10_ *K*_*aff*⁡_ = 12.7 (24), respectively, are in good agreement with experimental values, 5.6 [18] and 14 [24], respectively which lends confidence to other calculated free energy changes. 

At the present level of theory, Cu(II) species are predicted to be pentacoordinated in a square pyramidal configuration. The one exception we found is in the case of **7** (Figure 3), the proposed model for Component II of A*β*. In **7**, two carbonyl oxygen atoms, of Ala2 and His13, occupy nearly equivalent sites in the equatorial plane of a trigonal bipyramid. On the other hand, the predominant configuration at Cu(I) in water is linear dicoordination, with the exception of Cu(H_2_O)_3_^+^ and the possible exception of Cu(I) with all three His residues attached (modeled by 6^**I**^(**Im**) (Figure 4)) which are T-shaped structures.

It was found that Asp1 forms stable complexes with Cu(II) and *two* His residues, either the pair His6, His13, or the pair His6, His14, but not His13His14, both modeled by **2a**(**Im**)_2_ (Figure 1). Complexes involving the deprotonated amide of Ala2 are substantially less stable. The complex, **2a**(**Im**)_2_, represents the bonding configuration of Cu(II) in Component Ia and Component Ib of A*β*. Each has 3N1O square planar coordination with tridentate attachment of Asp1. The –NH_2_ and −CO_2_^−^ groups contribute one of the N ligands and the O ligand in the equatorial plane, while the carbonyl O of Asp1 occupies the axial position. 

The N-terminus does not attach to Cu(II) if His6 and His13His14 are already attached. Structure **7** is the best candidate for Component II. The assignment of **7** as a model for Component II is in agreement with the findings of Barnham and coworkers [12]. Hureau and Faller and coworkers suggested that the amide NH of Ala2 is deprotonated at higher pH and proposed a structure for Component II that is modeled by **4**(**Im**) (Figure 2) in our study [6, 7]. Our results suggest that the presence of the carboxylate group of Asp1 makes deprotonation of the amide group very improbable near physiological pH. Rather, we propose that deprotonation of a protonated His residue of either His13 or His14 facilitates the formation of Component II at the expense of Component I. 

The binding configuration of Cu(I) to A*β* is modeled by 6^**I**^ (Figure 4). The Cu(I) is linearly dicoordinated to His13His14 through the N_*δ*_ nitrogen atoms of the imidazole groups. Weak coordination of His6, as in 6^**I**^(**Im**), is possible in water as an endergonic process. 

Our calculations support in full the redox scheme for Cu/A*β*(1-16) proposed by Balland et al., which requires preorganization steps for both oxidized and reduced species [47]. The sequence of steps for reduction is described by the reverse of (15) and (27), and (36), which we repeat here for clarity:

(15′) $$\begin{array}{cc} 7+{\text{H}}_{2}\text{O}\to 6\left(Im\right)\left({\text{H}}_{2}\text{O}\right)+\text{NMA}; & \\ \Delta G_{\left(15^{\prime }\right)}=20\;\text{kJ}\;{\text{mol}}^{-1};\;\left[22\;\text{kJ}\;{\text{mol}}^{-1}\right], & \end{array}$$

(36′) $$\begin{array}{cc} & 6\left(Im\right)\left({\text{H}}_{2}\text{O}\right)+\;{\text{e}}^{-}\to 6^{I}\left(Im\right)+{\text{H}}_{2}\text{O};\\ & E^{{}^{\circ}}\left(6\left(Im\right)\left({\text{H}}_{2}\text{O}\right)/6I\left(Im\right),{\text{H}}_{2}\text{O}\right)=0.31\;\text{V};\;\left[0.30\;\text{V}\right], \end{array}$$

(27′) $$\begin{array}{cc} & 6^{I}\left(Im\right)\to 6^{I}+Im;\\ & \Delta G_{\left(27^{\prime }\right)}=-10\;\text{kJ}\;{\text{mol}}^{-1};\;\left[-16\;\text{kJ}\;{\text{mol}}^{-1}\right]. \end{array}$$

The experimental values derived from the data of Balland et al. are given in square parentheses. The reoxidation occurs by the exact reverse sequence.

## Supplementary Material

Table S1. B3LYP/6-31*+*G(d) structures of all species discussed in the paper and displayed in Figures 1–3.
Table S2. Primary properties calculated at the B3LYP/SB optimized structures of all species: SB = 6-31*+*G(d); LB = 6-311*+*(2df,2p).
Table S3. Relative energies at 298 K of species discussed in the text: enthalpies based on B3LYP/LB energies.

## Figures and Tables

**Table 1:** Relative energies at 298 K of species discussed in the text: enthalpies based on MP2/LB energies^a^.

Process	Eqn. no.	Δ*H*_(g)_ (kJ mol^−1^)	−*T*Δ*S*_(g)_ (kJ mol^−1^)	Δ*G*_(g)_ (kJ mol^−1^)	ΔΔ*G*_solv_ (kJ mol^−1^)	Δ*G*_(aq)_ (kJ mol^−1^)
1 + Cu(H_2_O)_5_^2+^ → 2**a**(H_2_O)_2_ + 3H_2_O + H^+^	(5a)	480.8	−68.6	412.2	−390.2	−18.0
1 + Cu(H_2_O)_5_^2+^ → 2**b**(H_2_O)_2_ + 3H_2_O + H^+^	(5b)	533.6	−76.1	457.6	−403.2	14.3
1 + Cu(H_2_O)_5_^2+^ → 2**c**(H_2_O)_2_ + 3H_2_O + H^+^	(5c)	519.3	−68.8	450.5	−381.4	29.1
3 + Cu(H_2_O)_5_ → 4(H_2_O) + 4H_2_O + H^+^	(6)	464.3	−90.6	373.6	−323.4	10.2
**I** **m** + Cu(H_2_O)_5_^2+^ → Cu(**I** **m**)(H_2_O)_4_^2+^ + H_2_O	(7a)	−157.3	6.5	−150.8	150.3	−0.5
2**I** **m** + Cu(H_2_O)_5_^2+^ → Cu(**I** **m**)_2_(H_2_O)_3_^2+^ + 2H_2_O	(7b)	−298.4	14.3	−284.1	273.2	−10.9
2**a**(H_2_O)_2_ + **I** **m** → 2**a**(H_2_O) + H_2_O	(8a)	−105.0	3.9	−101.1	77.0	−24.1
1 + Cu(**I** **m**)(H_2_O)_4_^2+^ → 2**a**(**I** **m**)(H_2_O) + 3H_2_O + H^+^	(8b)	533.1	−71.2	461.9	−463.5	−41.6
2**a**(**I** **m**)(H_2_O) + **I** **m** → 2**a**(**I** **m**)_2_ + H_2_O	(9a)	−85.5	6.1	−79.4	68.4	−11.0
1 + Cu(**I** **m**)_2_(H_2_O)_3_^2+^ → 2**a**(**I** **m**)_2_ + 3H_2_O + H^+^	(9b)	588.7	−72.9	515.8	−517.9	−42.2
4(H_2_O) + **I** **m** → 4(**I** **m**) + H_2_O	(10a)	−89.8	3.1	−86.7	66.2	−20.5
3 + Cu(H_2_O)_5_^2+^ + **I** **m** → 4(**I** **m**) + 5H_2_O + H^+^	(10b)	374.5	−87.5	287.0	−257.3	−10.3
4(**I** **m**) + **I** **m** → 4(**I** **m**)_2_	(11)	−105.6	35.9	−69.7	76.6	6.9
4(**I** **m**) + **I** **m** + 1 → 2**a**(**I** **m**)_2_ + 3	(12)	−84.1	28.8	−55.3	12.5	−42.8
5 + Cu(H_2_O)_5_^2+^ → 6(H_2_O)_2_ + 3H_2_O	(13)	−300.3	−29.5	−329.8	293.8	−36.0
6(H_2_O)_2_ + **I** **m** → 6(**I** **m**)(H_2_O) + H_2_O	(14)	−121.9	9.6	−112.2	99.7	−12.5
6(**I** **m**)(H_2_O) + NMA → 7 + H_2_O	(15)	68.5	−3.7	64.8	−85.4	−20.5
1 + 6(H_2_O)_2_ → 8**a** + 2H_2_O + H^+^	(16a)	623.3	−44.8	578.4	−535.6	2.8
2**a**(H_2_O)_2_ + 5 → 8**a** + 2H_2_O	(16b)	−157.9	−5.7	−163.6	148.4	−15.2
1 + 6(**I** **m**)(H_2_O) → 9 + H_2_O + H^+^	(17a)	608.0	−8.4	605.8	−543.8	22.0
9 → 8**a** + **I** **m**	(17b)	130.9	−46.0	84.8	−91.5	−6.7
9 + H_2_O + H^+^ → 5 + 3**a**(**I** **m**)H_2_O	(17c)	−183.7	36.4	−147.3	162.9	−24.4
1 + Cu(H_2_O)_3_^+^ → 2^**I**^(H_2_O) + 2H_2_O + H^+^	(18)	869.3	−40.1	829.2	−783.9	5.3
**I** **m** + Cu(H_2_O)_3_^+^ → Cu(**I** **m**)(H_2_O)^+^ + 2H_2_O	(19)	−73.2	−16.6	−89.8	73.5	−16.3
**I** **m** + Cu(**I** **m**)(H_2_O)^+^ → Cu(**I** **m**)_2_^+^ + H_2_O	(20a)	−120.1	8.5	−111.6	84.2	−27.4
5 + Cu(**I** **m**)(H_2_O)^+^ → Cu(**I** **m**)(H_13_H_14_)^+^ + H_2_O	(20b)	−105.1	−5.2	−110.4	67.0	−43.4
5 + Cu(**I** **m**)(H_2_O)^+^ → Cu(**I** **m**)(H_14_H_13_)^+^ + H_2_O	(20c)	−135.9	2.7	−133.2	102.2	−31.1
1 + Cu(**I** **m**)(H_2_O)^+^ → 2^**I**^(**I** **m**) + H_2_O + H^+^	(21)	854.4	−8.9	845.5	−808.5	−3.0
2^**I**^(**I** **m**) + **I** **m** → 2^**I**^(**I** **m**)_2_	(22)	−71.6	33.6	−38.1	60.8	22.7
1 + Cu(**I** **m**)_2_^+^ → 2^**I**^(**I** **m**)_2_ + H^+^	(23)	902.9	16.2	919.1	−831.9	47.2
5 + Cu(H_2_O)_3_^+^ → 6^**I**^ + 3H_2_O	(24)	−176.3	−23.4	−199.7	126.9	−72.8
1 + 6^**I**^ → 8**a** ^**I**^ + H^+^	(25a)	993.0	−7.1	985.9	−881.3	64.6
1 + 6^**I**^ → 8**b** ^**I**^ + H^+^	(25b)	940.3	17.0	957.3	−853.9	63.4
NMA + 6^**I**^ → 7^**I**^	(26)	−59.2	26.0	−33.2	58.2	25.0
**I** **m** + 6^**I**^ → 6^**I**^(**I** **m**)	(27)	−74.2	27.9	−46.3	56.4	10.1
2**a**(H_2_O)_2_ + e^−^ → 2**a** ^**I**^(H_2_O) + H_2_O	(28)	−641.7	−42.0	−683.7	215.9	−467.8^b^
2**a**(**I** **m**)(H_2_O) + e^−^ → 2^**I**^(**I** **m**) + H_2_O	(29)	−624.7	−31.4	−656.1	187.8	−468.3^b^
2**a**(**I** **m**)_2_ + 5 + e^−^ + H^+^ → 1 + Cu(**I** **m**)(H_13_H_14_)^+^	(30a)	−74.2	27.9	−46.3	56.4	10.1^b^
2**a**(**I** **m**)_2_ + 5 + e^−^ + H^+^ → 1 + Cu(**I** **m**)(H_14_H_13_)^+^	(30b)	−1529.6	−25.9	−1555.4	1030.0	−525.4^b^
2**a**(**I** **m**)_2_ + 5 + e^−^ + H^+^ → 1 + 6^**I**^(**I** **m**) + **I** **m**	(31)	−1570.9	−7.4	−1578.3	1037.7	−540.6^b^
2**a**(**I** **m**)_2_ + 5 + e^−^ + H^+^ → 1 + 6^**I**^ + 2**I** **m**	(32)	−1496.7	−35.3	−1532.1	981.3	−550.8^b^
6(H_2_O)_2_ + e^−^ → 6^**I**^ + 2H_2_O	(33)	−906.1	−64.5	−970.6	442.7	−527.9^b^
6(**I** **m**)(H_2_O) + e^−^ → 6^**I**^ + **I** **m** + H_2_O	(34)	−784.2	−74.1	−858.4	343.0	−515.4^b^
7 + e^−^ → 6^**I**^ + **I** **m** + NMA	(35)	−715.7	−77.8	−793.5	257.6	−535.9^b^
6(**I** **m**)(H_2_O) + e^−^ → 6^**I**^(**I** **m**) + H_2_O	(36)	−858.4	−46.2	−904.6	399.4	−505.3^b^

^a^Numbered structures are presented in Figures 1–4. ^b^The enthalpy correction 57 kJ mol^−1^ has not been added.
