# Supplementary material for: On the Involvement of Copper Binding to the N-Terminus of the Amyloid Beta Peptide of Alzheimer's Disease: A Computational Study on Model Systems
Source: Int J Alzheimers Dis. 2011 Dec 1;2011:539762. doi: 10.4061/2011/539762 (PMC3235578; doi:10.4061/2011/539762)
Supplement: Supplementary file 1 — Table S1. B3LYP/6-31+G(d) structures of all species discussed in the paper and displayed in Figures 1–3. Table S2. Primary properties calculated at the B3LYP/SB optimized structures of all species: SB = 6-31+G(d); LB = 6-311+(2df,2p). Table S3. Relative energies at 298 K of species discussed in the text: enthalpies based on B3LYP/LB energies. [file 539762.f1.pdf]

Supporting Information

On the Involvement of Copper binding to the N-Terminus of the Amyloid Beta Peptide of Alzheimer's Disease: A Computational Study

by

Samira Azimi and Arvi Rauk

Table S1. B3LYP/6-31+G(d) structures of all species discussed in the paper and displayed in Figures 1 – 3.

1 - D1 (Asp1)_LB, 6-311+g(2df,2p), Energy(B3LYP/SB)= -531.752579

0 1

C	0.002155	0.327219	-0.397226
H	0.092284	0.069224	-1.460742
C	0.917003	-0.579447	0.448645
C	-1.457006	0.262755	0.067889
H	0.823059	-0.300457	1.509286
H	0.576633	-1.614846	0.357236
O	-1.857596	1.022872	0.964407
N	-2.215653	-0.670747	-0.522314
H	-1.806688	-1.243980	-1.270151
C	-3.597603	-0.922559	-0.125860
H	-4.048015	-1.594909	-0.858381
H	-3.644198	-1.386239	0.866293
H	-4.160315	0.015138	-0.100524
N	0.477478	1.741585	-0.260193
H	1.533737	1.672621	-0.341738
H	0.085182	2.362640	-0.984861
H	0.215807	2.126922	0.661464
C	2.418130	-0.501300	0.067896
O	2.853692	0.624274	-0.350873
O	3.104362	-1.537395	0.227916

2a$(H_2O)_2$ - DCu(II)$(H_2O)_2^+$_LB, 6-311+g(2df,2p), Energy(B3LYP/SB)= -2324.213653

1 2

C	-1.195020	0.310615	1.302895
H	-1.939216	0.220490	2.105272
C	-1.114954	1.792316	0.881093
C	-1.598429	-0.614213	0.135608
H	-2.101552	2.139461	0.561912
H	-0.860832	2.391526	1.767047
O	-0.723399	-1.250911	-0.487012
N	-2.893609	-0.671557	-0.179475
H	-3.558016	-0.149864	0.379348
C	-3.401540	-1.447996	-1.316570
H	-4.452811	-1.196603	-1.460729

H	-2.839067	-1.197322	-2.218811
H	-3.307506	-2.520340	-1.121858
N	0.131994	-0.152879	1.791593
H	0.510865	0.520191	2.459764
H	0.058260	-1.048673	2.277966
C	-0.117705	2.185846	-0.227189
O	-0.328892	3.179734	-0.891988
O	0.980089	1.459787	-0.372442
Cu	1.308101	-0.320787	0.126234
O	1.676230	-2.328030	0.407742
O	2.746548	-0.130401	-1.349397
H	0.931773	-2.753531	-0.059902
H	2.489675	-2.764874	0.102009
H	3.708013	-0.144544	-1.204234
H	2.518080	0.777116	-1.643628

2b$(H_2O)_2$ - $DCu(II)(H_2O)_2^+$_LB, 6-311+g(2df,2p), Energy(B3LYP/SB)= -2324.20127

1 2

C	1.211618	0.174388	-0.447138
H	1.274805	0.599176	-1.454280
C	0.854184	1.276401	0.566214
C	2.540785	-0.516113	-0.042774
H	0.695913	0.821140	1.554072
H	1.689879	1.973626	0.665769
O	2.481407	-1.589981	0.565836
N	3.682351	0.112449	-0.360111
H	3.640805	0.994808	-0.854633
C	4.996398	-0.402208	0.038524
H	5.762709	0.144722	-0.512542
H	5.155400	-0.271132	1.113373
H	5.062892	-1.465610	-0.201185
N	0.181225	-0.915854	-0.461978
H	0.216609	-1.382715	-1.372625
H	0.566054	-1.613165	0.201908
C	-0.376577	2.099435	0.179978
O	-1.491852	1.416820	-0.079779
O	-0.353440	3.310721	0.127652
Cu	-1.710697	-0.427727	-0.084603
O	-3.711896	0.087998	-0.005251
H	-3.641118	1.062506	0.069944
H	-4.326215	-0.090272	-0.738995
O	-2.397789	-2.346589	0.241085
H	-3.230218	-2.426045	0.740049
H	-1.872218	-3.142302	0.432096

2c$(H_2O)_2$ - $DCu(II)(H_2O)_2^+$_LB, 6-311+g(2df,2p), Energy(B3LYP/SB)= -2324.180325

1 2

C	-1.956409	0.178379	-0.642970
H	-1.427142	0.342970	-1.582512
C	-1.913388	1.445915	0.221340
C	-1.456577	-1.105008	0.091916
H	-2.555645	2.219802	-0.220271
H	-2.295447	1.250824	1.230341
O	-2.355630	-1.924835	0.404555
N	-0.167911	-1.248697	0.366874
C	0.163562	-2.450778	1.158836
H	1.216057	-2.419719	1.450639
H	-0.445805	-2.490858	2.066970
H	-0.031054	-3.366648	0.587472
N	-3.398602	-0.175798	-0.966714
H	-3.556131	-0.374766	-1.958049
H	-3.512840	-1.080004	-0.395127
C	-0.524276	2.092875	0.345304
O	0.434494	1.563058	-0.400374
O	-0.375934	3.076434	1.041282
H	-4.070631	0.534904	-0.666142
Cu	1.288294	-0.097912	-0.130668
O	2.965704	1.122620	-0.226308
H	3.588266	1.201070	0.517699
H	2.481417	1.974945	-0.286477
O	2.650945	-1.601230	-0.552817
H	2.366341	-2.488246	-0.830994
H	3.413584	-1.350053	-1.102577

2a(**Im**)(H_2O) - DCu(II) (**Im**) (H_2O) $^+$_LB, 6-311+g(2df,2p), Energy(B3LYP/SB)= -2474.047467

1 2

C	1.69473800	0.45973600	1.41959900
H	2.06623100	1.03390500	2.27954100
C	2.69635800	-0.67329400	1.12448000
C	1.51854000	1.41588300	0.22185100
H	3.69056100	-0.25337300	0.94755800
H	2.79431300	-1.29639500	2.02481500
O	0.52167000	1.32406700	-0.51701000
N	2.49074400	2.31344300	0.01791900
H	3.27014700	2.34903900	0.66292200
C	2.49625800	3.22465900	-1.12982100
H	3.45087300	3.75201800	-1.14525500
H	2.37731300	2.65977600	-2.05773600
H	1.68229000	3.95115500	-1.04816900
N	0.35847000	-0.10940600	1.73336500
H	0.45569500	-0.86877300	2.40868900
H	-0.25197300	0.59304000	2.15296700
C	2.38615600	-1.62098600	-0.05318700
O	3.30229700	-2.23270200	-0.56979700

O	1.12783900	-1.76840600	-0.41464800
Cu	-0.43232700	-0.75205800	-0.07112200
O	-0.90225300	-1.60819200	-1.92795600
H	-1.65831900	-2.19786400	-2.08277000
H	-0.08315500	-2.13726100	-2.03326000
C	-3.38608000	-0.57435200	0.75690500
C	-4.46253500	0.24676100	0.55602800
C	-2.67044900	1.11358300	-0.42455800
H	-3.33852000	-1.51112300	1.29280700
H	-5.49290300	0.17466400	0.86913300
H	-4.53280900	2.09335200	-0.51906800
H	-2.04014400	1.78634700	-0.98784600
N	-2.27423700	-0.02211000	0.14092800
N	-3.98732600	1.30537500	-0.19139200

2b(**Im**)(H_2O) - DCu(II) (**Im**) (H_2O) $^+$_LB, 6-311+g(2df,2p), Energy(B3LYP/SB)= -2474.044391

1 2

C	2.193165	-0.046924	-1.257299
H	2.997437	-0.295401	-1.964770
C	1.999790	1.477370	-1.254206
C	2.585156	-0.682590	0.094731
H	2.959489	1.986611	-1.129016
H	1.634165	1.782513	-2.244751
O	2.085606	-1.770586	0.440121
N	3.512908	-0.054039	0.822863
H	3.824754	0.866323	0.540054
C	4.004629	-0.580915	2.099292
H	4.924366	-0.053982	2.356878
H	3.265001	-0.430231	2.892109
H	4.208997	-1.648493	1.999216
N	0.936603	-0.710900	-1.708121
H	0.708775	-0.377498	-2.647182
H	1.091647	-1.718687	-1.784927
C	1.030860	2.022755	-0.192811
O	1.330240	3.008504	0.458409
O	-0.126146	1.406233	-0.049293
Cu	-0.626139	-0.371446	-0.466394
O	-0.498287	-2.380422	0.097862
H	0.466172	-2.410205	0.339217
H	-0.999264	-2.682367	0.873458
C	-3.556671	-0.871724	-0.034528
C	-4.292354	0.981424	0.934615
C	-2.945803	1.086066	0.720707
H	-3.562129	-1.853790	-0.485352
H	-5.593576	-0.646533	0.449226
H	-5.005225	1.660664	1.376331
H	-2.262282	1.891974	0.940842

N	-2.498375	-0.078127	0.116923
N	-4.657456	-0.260013	0.450378

2a(**Im**)$_2$ - DCu(II) (**Im**)$_2^+$ _LB, 6-311+g(2df,2p), Energy(B3LYP/SB)= -2623.873195

1 2

C	2.330252	-1.225570	-0.779748
H	2.944698	-2.108122	-1.006987
C	3.047862	0.018647	-1.335551
C	2.115200	-1.157178	0.745295
H	4.036361	0.116705	-0.877955
H	3.232397	-0.137555	-2.408035
O	0.993398	-0.904001	1.215541
N	3.191368	-1.375374	1.515799
H	4.081139	-1.568493	1.073876
C	3.149758	-1.261503	2.975044
H	4.130890	-1.529949	3.369204
H	2.904745	-0.238071	3.274022
H	2.396570	-1.939242	3.385393
N	0.991936	-1.363523	-1.406777
H	1.070627	-1.235265	-2.416266
H	0.609070	-2.296194	-1.249267
C	2.333565	1.380315	-1.196559
O	3.013430	2.394774	-1.248874
O	1.029378	1.392915	-1.093257
Cu	-0.254565	0.073310	-0.546543
C	-0.942244	2.857772	0.206379
C	-2.765672	2.821455	1.472518
C	-2.452704	1.549910	1.076596
H	-0.074917	3.210471	-0.332280
H	-1.730486	4.636231	1.005091
H	-3.560982	3.211223	2.089195
H	-2.954537	0.624666	1.313763
N	-1.316166	1.584746	0.286912
N	-1.798267	3.630872	0.908969
H	-4.553609	-2.621568	-1.177573
H	-3.575872	-3.703918	0.920291
H	-1.206734	-2.311819	1.362765
N	-1.806523	-1.245306	-0.407054
C	-3.111138	-2.894592	0.377987
C	-1.945447	-2.204706	0.582194
N	-3.679324	-2.340074	-0.751074
C	-2.868732	-1.351340	-1.195372
H	-3.082006	-0.743257	-2.062881

2b(**Im**)$_2$ - DCu(II) (**Im**)$_2^+$ _LB, 6-311+g(2df,2p), Energy(B3LYP/SB)= -2623.865365

1 2

C	-2.138526	-1.677505	-0.585608
H	-2.844629	-2.285338	-1.166972
C	-2.015403	-2.280193	0.836727
C	-2.614097	-0.219945	-0.618525
H	-2.882496	-1.984094	1.438686
H	-2.018002	-3.372223	0.768191
O	-1.804592	0.700188	-0.890405
N	-3.901416	0.029701	-0.383000
H	-4.521909	-0.750904	-0.207301
C	-4.458733	1.385048	-0.355267
H	-5.491986	1.324117	-0.011408
H	-3.881400	2.010270	0.330001
H	-4.432578	1.831866	-1.353335
N	-0.799928	-1.668247	-1.240246
H	-0.884026	-1.630937	-2.257049
C	-0.716099	-1.810001	1.542405
O	0.026484	-2.668629	2.036194
O	-0.495484	-0.543985	1.476807
H	-0.284526	-2.519719	-1.011729
Cu	0.116697	0.062193	-0.503284
C	1.310868	4.022823	0.255852
C	0.684332	2.200025	1.358605
H	1.627361	5.044743	0.114227
H	1.153321	3.975597	2.388742
H	0.416864	1.524241	2.159274
N	0.675491	1.884053	0.069577
N	1.066148	3.488354	1.505968
C	1.062977	3.010476	-0.631486
H	1.127271	3.022368	-1.709350
C	2.445415	-1.482802	0.427870
C	4.122086	-1.180360	-1.001207
C	3.017944	-0.501832	-1.440105
H	1.873648	-1.842896	1.279531
H	4.316988	-2.378773	0.761460
H	5.116552	-1.279581	-1.408507
H	2.905633	0.108364	-2.324102
N	1.980489	-0.699736	-0.544286
N	3.736909	-1.790653	0.176063

3 - Asp1Ala2: b3lyp/6-31+g(d) scrf-iefpcm: OPT: -779.167087628

```
0 1
 C          -1.794005   -0.570140   -0.261491
 H          -1.767289   -0.494714   -1.356330
 C          -2.251486    0.765059    0.356387
 C          -0.435282   -1.009931    0.297367
 H          -2.231219    0.686054    1.453803
 H          -1.536765    1.545208    0.078245
```

O	-0.386296	-1.656986	1.354577
N	0.634145	-0.604949	-0.406792
C	2.013252	-0.862752	0.003311
C	-3.675192	1.220613	-0.055234
O	-3.904479	2.451504	-0.039916
O	-4.523326	0.307989	-0.342285
O	2.722814	1.037598	-1.317209
C	2.840888	0.405208	-0.254682
N	3.710635	0.746281	0.711279
H	3.726027	0.205748	1.582345
C	4.618901	1.880168	0.577362
H	5.247433	1.767469	-0.312234
H	4.061474	2.819529	0.491995
H	5.254045	1.918546	1.464441
H	1.993292	-1.089144	1.074694
C	2.612884	-2.054847	-0.764965
H	2.617216	-1.856839	-1.842333
H	3.642426	-2.242279	-0.439405
H	2.018962	-2.954611	-0.574184
H	0.489618	-0.136867	-1.310561
N	-2.802621	-1.625950	0.069737
H	-2.716923	-1.918499	1.056659
H	-3.739526	-1.152705	-0.094862
H	-2.703347	-2.465679	-0.521509

4(H_2O) - D1A2_Cu2_W: b3lyp/6-31+g(d) : OPT: -2495.12810223

1 2

C	-2.070062	0.617642	-1.261378
H	-2.566655	1.065808	-2.127542
C	-3.108336	0.575985	-0.126181
C	-0.880404	1.546783	-0.896402
H	-3.381807	1.604002	0.130274
H	-4.035135	0.097862	-0.477539
O	-1.031687	2.768042	-0.902120
N	0.226338	0.858326	-0.563192
C	1.459537	1.484507	-0.113634
N	-1.566569	-0.751099	-1.610958
H	-2.285014	-1.464846	-1.482333
H	-1.304356	-0.777005	-2.597665
C	-2.703862	-0.159489	1.137749
O	-3.422341	0.241252	2.187748
O	-1.864601	-1.050895	1.217135
Cu	0.116954	-1.020497	-0.470348
O	2.017915	-0.877649	0.100609
O	-0.075919	-2.996804	-0.027550
H	0.738962	-3.465671	0.223983
H	-0.689268	-3.063829	0.727651

```
 H           -3.173984   -0.289573    2.970626
 C            2.436488    0.321798    0.087670
 N            3.724781    0.580807    0.278519
 H            4.022562    1.548551    0.258161
 C            4.734345   -0.453917    0.522780
 H            4.511556   -0.993202    1.447575
 H            4.757745   -1.163087   -0.308734
 H            5.706687    0.031542    0.612074
 H            1.852775    2.154703   -0.892727
 C            1.299992    2.290299    1.195771
 H            0.955895    1.643073    2.009904
 H            2.246662    2.753380    1.496047
 H            0.566756    3.083260    1.034925
```

4(H_2O)(CO_2^-) - D1A2_Cu2_W_Om: b3lyp/6-31+g(d) : almost OPT(10^-6): -2494.72328015

```
0 2
 C       -1.950883    1.238031   -1.062767
 H       -2.389786    2.026577   -1.687511
 C       -2.994767    0.893161    0.021105
 C       -0.657658    1.862654   -0.490947
 H       -3.045388    1.727157    0.731950
 H       -3.987908    0.848547   -0.442853
 O       -0.538980    3.094761   -0.396013
 N        0.267039    0.952428   -0.155975
 C        1.577170    1.333177    0.312226
 N       -1.616255    0.028132   -1.863138
 H       -2.466795   -0.473226   -2.121176
 H       -1.158425    0.299578   -2.732716
 C       -2.876945   -0.406569    0.853076
 O       -3.863905   -0.757687    1.492782
 O       -1.754942   -1.080673    0.852596
 Cu      -0.121617   -0.906013   -0.218177
 O        1.965015   -1.012601   -0.199113
 O       -0.419849   -2.946435   -0.261096
 H        0.253746   -3.479105    0.192003
 H       -1.196589   -2.871616    0.342388
 C        2.466260    0.117699    0.031349
 N        3.796365    0.277177    0.055481
 H        4.159652    1.202153    0.243132
 C        4.736617   -0.821414   -0.150171
 H        4.668318   -1.550404    0.663906
 H        4.522366   -1.330803   -1.093783
 H        5.747460   -0.410642   -0.182401
 H        1.945643    2.197762   -0.261542
 C        1.606896    1.701174    1.813806
 H        1.252426    0.862870    2.423474
 H        2.613442    1.981830    2.149476
```

```
 H        0.939850    2.553147    1.968910
```

4(Im) - D1A2_Cu2_Im: b3lyp/6-31+g(d) : OPT(10^-7): -2644.96232341

```
1 2
 C         -2.172384   -0.411850   -1.190791
 H         -2.048256   -0.450844   -2.279170
 C         -3.640895   -0.737408   -0.878242
 C         -1.806238    1.034659   -0.779676
 H         -4.265888    0.061723   -1.286623
 H         -3.948141   -1.669642   -1.372016
 O         -2.622362    1.948811   -0.913205
 N         -0.535904    1.148005   -0.351599
 C          0.020452    2.450094   -0.025883
 N         -1.187508   -1.372077   -0.601364
 H         -1.550516   -1.647506    0.320778
 H         -1.109855   -2.210521   -1.177159
 C         -3.932172   -0.891899    0.599982
 O         -5.221590   -0.687896    0.889926
 O         -3.103792   -1.205778    1.443377
 Cu         0.581198   -0.365662   -0.226832
 O          2.017351    1.068686    0.021148
 H         -5.353443   -0.838162    1.847016
 C          1.532275    2.239000    0.016818
 N          2.332183    3.301188    0.073856
 H          1.902838    4.218021    0.060488
 C          3.790757    3.217145    0.163465
 H          4.088412    2.699763    1.080102
 H          4.194507    2.680808   -0.699838
 H          4.193006    4.230839    0.176089
 H         -0.223281    3.171119   -0.820428
 C         -0.499579    3.008673    1.317742
 H         -0.250957    2.331324    2.141841
 H         -0.078031    3.996631    1.535868
 H         -1.585580    3.107882    1.255302
 N          1.846908   -1.873239   -0.006200
 N          3.762487   -2.923787    0.200176
 C          1.596109   -3.225042    0.165172
 C          3.168245   -1.724999    0.022143
 C          2.782928   -3.891669    0.295229
 H          0.593942   -3.624753    0.198256
 H          3.011763   -4.935390    0.446611
 H          4.761140   -3.081989    0.255885
 H          3.692596   -0.786549   -0.074676
```

4(**Im**)(CO_2^-) - D1A2_Cu2_Im_Om: b3lyp/6-31+g(d) : OPT (10^-7): -2644.53790573

```
0 2
```

C	0.237204	-2.601666	-1.224496
H	0.050101	-3.421727	-1.930318
C	1.182038	-3.152376	-0.138146
C	-1.156792	-2.232168	-0.674446
H	0.631269	-3.891233	0.457026
H	1.990375	-3.716134	-0.621653
O	-2.089086	-3.053612	-0.734588
N	-1.230098	-0.992232	-0.171741
C	-2.493943	-0.479751	0.296688
N	0.853179	-1.428026	-1.896528
H	1.831198	-1.620703	-2.114270
H	0.383304	-1.246830	-2.782978
C	1.883716	-2.203812	0.862453
O	2.802828	-2.685720	1.528114
O	1.511733	-0.962754	0.979926
Cu	0.297328	0.151022	-0.060699
O	-1.338645	1.616708	-0.110713
C	-2.430441	1.033997	0.082362
N	-3.581278	1.727581	0.131149
H	-4.439947	1.211154	0.266359
C	-3.646572	3.177664	-0.008776
H	-3.007872	3.662802	0.735578
H	-3.318775	3.488512	-1.006523
H	-4.680185	3.495363	0.142988
H	-3.312565	-0.902578	-0.305971
C	-2.769165	-0.814280	1.781530
H	-1.974359	-0.415191	2.421113
H	-3.732766	-0.415011	2.124071
H	-2.788425	-1.902254	1.888429
N	1.651740	1.628734	-0.081257
N	2.648350	3.561738	-0.375884
C	2.948055	1.524683	0.387023
C	1.496568	2.863672	-0.530744
C	3.583309	2.724090	0.208902
H	3.292003	0.600430	0.828157
H	4.583966	3.053332	0.442880
H	2.791882	4.528013	-0.635182
H	0.586520	3.270577	-0.945628

4(Im)$_2$ - D1A2_Cu2_Im2: b3lyp/6-31+g(d) : almost OPT(10^-6): -2871.21012354

1 2

C	2.225471	-0.533063	1.074923
H	1.903573	-0.602826	2.120541
C	3.703714	-0.946113	1.016684
C	2.034369	0.947597	0.661484
H	4.291372	-0.223528	1.589297
H	3.852833	-1.928479	1.486492

O	2.903758	1.786589	0.929244
N	0.853779	1.169798	0.067535
C	0.496232	2.502020	-0.384242
N	1.306051	-1.401466	0.280659
H	1.785264	-1.634571	-0.596176
H	1.115671	-2.269213	0.781282
C	4.258950	-1.028812	-0.389383
O	5.593862	-0.911512	-0.417759
O	3.591502	-1.220013	-1.394842
Cu	-0.378177	-0.270889	-0.208557
O	-1.556589	1.312799	-0.902518
H	5.890244	-1.005090	-1.344440
C	-0.983447	2.425502	-0.756871
N	-1.650837	3.567287	-0.948655
H	-1.146416	4.436739	-0.831198
C	-3.052920	3.625839	-1.357613
H	-3.177375	3.251116	-2.378848
H	-3.667392	3.026372	-0.680742
H	-3.381506	4.665409	-1.316061
H	0.629308	3.232519	0.428932
C	1.334297	2.970836	-1.596672
H	1.207541	2.284091	-2.440569
H	1.051238	3.979894	-1.918649
H	2.387275	2.986775	-1.307262
N	-1.507582	-1.660883	-1.163683
N	-3.068595	-2.454402	-2.491545
C	-1.345337	-3.030549	-1.273952
C	-2.556659	-1.343693	-1.912272
C	-2.311345	-3.539776	-2.099590
H	-0.550632	-3.556546	-0.766625
H	-2.518799	-4.543650	-2.437190
H	-3.863719	-2.479108	-3.117432
H	-2.945059	-0.345403	-2.046974
C	-1.131985	0.396406	2.929252
C	-1.932654	0.089331	3.999227
C	-2.391548	-1.235924	2.285212
N	-1.425365	-0.436282	1.865024
N	-2.729298	-0.953320	3.571191
H	-0.370093	1.159069	2.853596
H	-2.007630	0.502610	4.993668
H	-3.439829	-1.422868	4.117625
H	-2.868891	-2.013163	1.705086

4(Im)$_2$(CO_2^-) - D1A2_Cu2_Im2_Om: b3lyp/6-31+g(d) : almost OPT (10^-7): -2870.76148468

0 2

C	1.994430	-0.240295	-1.971340
H	1.948459	-0.399854	-3.054643

```
C          3.442540    0.190187   -1.590203
C          1.625723   -1.584976   -1.315719
H          4.059655   -0.706667   -1.503759
H          3.840057    0.807261   -2.405505
O          2.147943   -2.643042   -1.695154
N          0.729963   -1.464035   -0.315738
C          0.421514   -2.596546    0.529658
N          1.018083    0.823032   -1.581836
H          1.575772    1.666858   -1.308489
H          0.364493    1.032097   -2.333229
C          3.520369    1.038746   -0.284410
O          4.114668    0.575783    0.700808
O          2.923152    2.178677   -0.372784
Cu        -0.049286    0.231023    0.053003
O         -1.087503   -0.944764    1.481783
C         -0.732824   -2.145783    1.423207
N         -1.354837   -3.075129    2.169727
H         -1.014163   -4.025831    2.122223
C         -2.411626   -2.752881    3.121462
H         -2.036949   -2.097505    3.914971
H         -3.239601   -2.248200    2.614723
H         -2.775158   -3.682666    3.563858
H          0.092151   -3.461521   -0.070456
C          1.628152   -3.039167    1.393134
H          1.972808   -2.211309    2.021290
H          1.379768   -3.897181    2.031591
H          2.441659   -3.328108    0.724613
N         -0.410139    1.963204    1.005707
N         -1.333721    3.429980    2.352854
C          0.524957    2.954199    1.272136
C         -1.512264    2.271566    1.673052
C         -0.043885    3.874814    2.111018
H          1.530713    2.893171    0.852222
H          0.344710    4.778415    2.554933
H         -2.016846    3.874898    2.950109
H         -2.417464    1.683071    1.701357
C         -2.568889   -0.786885   -2.119180
C         -3.621442   -0.467917   -2.940355
C         -2.950630    1.309026   -1.801510
N         -2.159916    0.326589   -1.413366
N         -3.855842    0.876429   -2.725476
H         -2.070801   -1.738731   -1.999706
H         -4.203787   -1.049670   -3.639020
H         -4.559327    1.440268   -3.183054
H         -2.904819    2.333276   -1.457122
```

5 - L_LB: B3LYP/6-311+g(2df,2p), Energy(B3LYP/SB)= -777.24067

```
0 1
```

C	3.725769	-0.553863	0.307272
N	3.834962	0.693727	-0.271776
C	5.056842	0.762682	-0.873443
N	5.746519	-0.351651	-0.714739
C	4.922622	-1.177697	0.019614
O	1.160448	1.438325	0.136992
C	0.515349	0.380480	0.198331
N	-0.837771	0.360036	0.164394
C	-1.649335	1.557902	-0.022032
C	-3.004894	1.429663	0.694022
C	-3.879624	0.330320	0.160656
C	-5.167599	0.402920	-0.317149
N	-5.508224	-0.888887	-0.673168
C	-4.432186	-1.681944	-0.407837
N	-3.435980	-0.982431	0.096079
C	2.521148	-1.000095	1.079497
C	1.181520	-0.992041	0.304839
H	0.482874	-1.690904	0.781500
H	2.404750	-0.399000	1.992370
H	2.712400	-2.027491	1.409603
H	5.392049	1.640026	-1.410867
H	-1.340227	-0.524702	0.172257
H	-1.808047	1.746184	-1.094013
H	-2.820329	1.270009	1.765330
H	-3.534847	2.385096	0.600700
H	-4.428160	-2.746808	-0.597592
H	-6.392222	-1.189240	-1.060138
H	-5.853245	1.229729	-0.430806
H	5.236779	-2.173624	0.306128
H	1.347158	-1.362603	-0.716354
H	-1.085984	2.404200	0.379023
H	3.069237	1.367002	-0.261213

6$(H_2O)_2$ - L Cu(II) $(H_2O)_2^{2+}$_LB: B3LYP/6-311+g(2df,2p), Energy(B3LYP/SB)= -2569.99388

2 2

C	-3.882486	-0.480816	-0.618174
C	-2.789854	-0.148316	0.142746
N	-1.694546	-0.874696	-0.340549
C	-2.135845	-1.608403	-1.365251
N	-3.448231	-1.389866	-1.557297
C	-2.744906	0.782756	1.322134
C	-2.538593	2.283332	1.014795
Cu	0.241934	-0.789396	0.167764
N	2.170877	-0.410184	-0.056604
C	3.234685	-0.954193	0.530307
N	4.337879	-0.254190	0.203553
C	3.970657	0.788070	-0.621572
C	2.610635	0.698103	-0.777966
C	1.690217	1.576583	-1.577991
C	0.872544	2.628778	-0.781419
O	0.022076	1.044967	0.852063
C	-0.206703	2.051938	0.112473
N	-1.397560	2.639243	0.143770
H	0.439527	3.343457	-1.489129

H	1.008560	0.956110	-2.175377
H	2.296366	2.129759	-2.302202
H	3.230821	-1.810527	1.189377
H	5.281657	-0.463677	0.511188
H	-1.513957	3.483321	-0.408362
H	-3.422473	2.681737	0.509707
H	-1.995757	0.439066	2.035196
H	-3.711996	0.728785	1.835755
H	-1.542556	-2.284713	-1.961808
H	-4.021963	-1.834224	-2.266098
H	-4.914407	-0.167005	-0.557894
H	4.693437	1.481662	-1.025794
H	1.549975	3.196592	-0.129289
H	-2.431348	2.821808	1.963716
O	0.506079	-2.756191	-0.556206
H	1.389874	-2.987598	-0.892422
H	0.198036	-3.518828	-0.035190
O	0.424608	-1.536080	2.379308
H	-0.079415	-2.183235	2.901571
H	0.646394	-0.820714	2.999990

6(**Im**)(H_2O) - LCu(II)(**Im**)(H_2O)$^+$_LB: B3LYP/6-311+g(2df,2p), Energy(B3LYP/SB)= -2719.83236

2 2

C	3.407421	1.986458	-0.733519
C	2.667319	1.156555	0.071641
N	1.338394	1.206775	-0.360365
C	1.301401	2.043779	-1.395863
N	2.532145	2.526861	-1.650086
C	3.138697	0.354064	1.251057
C	3.739677	-1.036002	0.940472
Cu	-0.305676	0.149083	0.244491
N	-1.541238	1.711856	-0.004269
N	-1.709554	-1.266609	-0.034571
C	-2.859988	-1.444601	0.604623
N	-3.385576	-2.642681	0.276340
C	-2.531709	-3.266245	-0.609659
C	-1.476638	-2.408656	-0.796345
C	-0.257131	-2.589100	-1.655314
C	1.028445	-3.060360	-0.926523
O	0.915672	-1.337562	0.779813
C	1.636969	-2.035493	0.010303
N	2.961507	-1.905879	0.035626
H	1.762429	-3.375103	-1.676050
H	-0.048932	-1.661793	-2.205304
H	-0.484545	-3.342563	-2.416095
H	-3.315320	-0.753353	1.298472
H	-4.260797	-3.019386	0.622683
H	3.503088	-2.533772	-0.549040
H	4.714626	-0.917865	0.460448
H	2.326217	0.262480	1.971636
H	3.939835	0.910633	1.751634
H	0.422455	2.318301	-1.959337
H	2.768600	3.188523	-2.380915

H	4.455578	2.247230	-0.718661
H	-2.748131	-4.238512	-1.027472
H	0.795992	-3.945174	-0.318477
H	3.901401	-1.566642	1.886239
C	-1.401360	2.930585	0.642672
C	-2.636505	1.799829	-0.755847
N	-3.194487	3.020609	-0.610969
C	-2.430252	3.753589	0.272999
H	-3.034562	1.024853	-1.393971
H	-4.035221	3.343330	-1.077398
H	-2.680237	4.765802	0.553934
H	-0.579960	3.127602	1.314989
O	-0.483433	0.306551	2.629612
H	-0.107030	-0.458275	3.095679
H	-0.855220	0.885417	3.315254

---

7 - LCu(II)(**Im**)(NMA)_LB: B3LYP/6-311+g(2df,2p), Energy(B3LYP/SB)= -2891.95984

2 2

C	-3.70321	1.83933	-0.76392
C	-2.90105	0.74905	-0.53131
N	-1.61851	1.21565	-0.24494
C	-1.6568	2.54197	-0.31157
N	-2.90323	2.95277	-0.6235
C	-3.31019	-0.69737	-0.57201
C	-3.10553	-1.42692	-1.93643
Cu	0.03354	0.12444	0.10688
N	-0.62446	-0.34365	2.02828
N	1.67895	-1.02996	0.32497
C	2.46849	-0.862	1.38298
N	3.51433	-1.70814	1.32598
C	3.39722	-2.4601	0.17745
C	2.25422	-2.03782	-0.45665
C	1.76447	-2.53333	-1.79064
C	0.34267	-3.11917	-1.84058
O	-0.42643	-0.82697	-1.71193
C	-0.72559	-2.0405	-1.82357
N	-2.0084	-2.40399	-1.93483
H	0.17275	-3.81867	-1.01134
H	2.46098	-3.31504	-2.11
H	1.83786	-1.7332	-2.53588
H	2.30807	-0.15633	2.18407
H	4.25689	-1.77873	2.01208
H	-2.23204	-3.38141	-2.0849
H	-4.00645	-1.98792	-2.19172
H	-4.37711	-0.73807	-0.32785
H	-2.80046	-1.25783	0.21907

H	-0.81815	3.1997	-0.14446
H	-3.19701	3.9155	-0.73971
H	-4.75249	1.91276	-1.00947
H	4.12348	-3.21154	-0.09521
H	0.23119	-3.70608	-2.76162
H	-2.92776	-0.70068	-2.73515
C	-0.53738	-1.56621	2.67723
C	-1.25613	0.47775	2.86126
N	-1.56921	-0.16784	4.00618
C	-1.12428	-1.46953	3.91013
H	-1.49773	1.51266	2.6713
H	-2.04644	0.23944	4.80241
H	-1.26382	-2.18962	4.70244
H	-0.06538	-2.42271	2.22058
O	1.24482	1.88784	-0.12718
C	2.04677	2.31578	-0.99965
C	2.21224	1.63249	-2.33521
C	2.78282	4.16139	0.48612
N	2.79835	3.39731	-0.7617
H	2.72969	0.67858	-2.18523
H	1.22899	1.41262	-2.76025
H	2.7878	2.22662	-3.04996
H	3.40164	3.7259	-1.50548
H	3.73628	4.68263	0.58835
H	1.97332	4.89949	0.48707
H	2.64872	3.48204	1.32941

8a - LDCu(II)$^{+}$ _LB: B3LYP/6-311+g(2df,2p), Energy(B3LYP/SB)= -2948.63962

1 2

C	0.932310	-3.072842	2.518297
C	0.585484	-2.546100	1.296640
N	0.672380	-1.161460	1.365545
C	1.055060	-0.864626	2.596055
N	1.230916	-1.993690	3.325903
C	0.098636	-3.251124	0.063034
C	1.159227	-3.698287	-0.965600
Cu	0.216338	0.441096	-0.135834
N	1.908742	1.645918	-0.124900
C	1.759867	2.941897	0.141282
N	2.927535	3.591481	-0.049086
C	3.876376	2.674784	-0.451040
C	3.237066	1.461192	-0.495250
C	3.863030	0.135774	-0.808326
C	3.261744	-0.640299	-2.012647
O	0.884576	-0.878059	-1.593907
C	2.014981	-1.419648	-1.643007

N	2.188750	-2.714472	-1.338819
H	4.025136	-1.324249	-2.400540
H	3.844312	-0.508278	0.081394
H	4.919923	0.319460	-1.027910
H	0.849198	3.417294	0.483166
H	3.071676	4.583578	0.089778
H	3.120458	-3.087818	-1.471163
H	1.712320	-4.554964	-0.570439
H	-0.638117	-2.617778	-0.432835
H	-0.430621	-4.165813	0.355926
H	1.190733	0.133943	2.987310
H	1.502930	-2.034883	4.299794
H	0.969886	-4.088917	2.882704
H	4.900471	2.951822	-0.652097
H	3.000725	0.052840	-2.817739
H	0.639896	-4.032066	-1.872412
O	-0.572229	1.662816	1.142241
C	-1.524206	2.500581	0.816014
O	-1.434072	3.716873	0.956108
C	-2.837033	1.904994	0.265229
H	-3.599262	2.121584	1.023581
H	-3.123537	2.471901	-0.630006
N	-1.636678	0.122328	-0.939866
H	-1.753994	-0.831979	-1.292869
H	-1.730039	0.708481	-1.774308
C	-2.767425	0.404491	-0.019743
H	-2.539098	-0.117883	0.917244
C	-4.031005	-0.278322	-0.596824
N	-5.238194	0.191969	-0.221260
O	-3.892703	-1.259382	-1.332724
H	-5.296179	1.025570	0.345741
C	-6.480476	-0.440424	-0.664406
H	-6.594625	-0.353989	-1.749499
H	-6.479379	-1.501228	-0.399615
H	-7.317176	0.056834	-0.170648

8b - LDCu(II)$^+$ _LB: B3LYP/6-311+g(2df,2p), Energy(B3LYP/SB)= -2948.63756

1 2

C	4.661990	-0.710166	0.494380
C	4.020023	-1.925750	0.563743
N	4.911569	-2.953804	0.320350
C	6.075463	-2.374144	0.115791
N	5.977974	-1.014902	0.209455
C	2.578089	-2.227686	0.841169
C	1.896870	-2.825000	-0.404695
Cu	-1.624078	0.300820	-0.169486
N	-3.440615	-0.556276	-0.051289

C	-4.503322	0.163431	0.306227
N	-5.608604	-0.603716	0.272764
C	-5.248447	-1.876680	-0.122880
C	-3.893332	-1.843592	-0.326925
C	-3.015178	-2.971063	-0.775052
C	-1.866252	-3.329897	0.202797
O	-0.526079	-1.381688	-0.387268
C	-0.570980	-2.615703	-0.120876
N	0.545297	-3.343662	-0.137506
H	-1.697997	-4.411639	0.189843
H	-2.600514	-2.759502	-1.769097
H	-3.650816	-3.853802	-0.894154
H	-4.473082	1.209656	0.572664
H	-6.543440	-0.289138	0.500990
H	0.464430	-4.327612	0.088476
H	1.821535	-2.080159	-1.200713
H	2.052829	-1.320450	1.159646
H	2.508977	-2.952159	1.663328
H	7.007978	-2.879605	-0.096265
H	6.735365	-0.352850	0.107933
H	4.309499	0.305594	0.604369
H	-5.967435	-2.675214	-0.227803
H	-2.150946	-3.070341	1.230683
H	2.491064	-3.662067	-0.778376
O	-2.432877	1.945334	0.314676
C	-1.889859	3.068633	0.741681
O	-2.564510	4.029603	1.073589
C	-0.360456	3.124475	0.813751
H	-0.079254	4.157017	1.034929
H	-0.007809	2.506355	1.651409
N	0.133458	1.187580	-0.659694
H	0.327040	0.930200	-1.630356
H	0.884599	0.729034	-0.129303
C	0.324002	2.649057	-0.479789
H	-0.121891	3.159058	-1.340912
C	1.842045	2.923840	-0.405843
N	2.250945	4.157945	-0.756684
O	2.604011	2.037075	-0.007315
H	1.566399	4.832453	-1.073334
C	3.647417	4.579370	-0.646335
H	3.990276	4.504885	0.389932
H	4.285587	3.951091	-1.274428
H	3.722902	5.615945	-0.978476

9 - LDCu(II) (**Im**) $^{+}$_LB , 6-311+g(2df,2p), Energy(B3LYP/SB)= -3174.89184

1 2

C 3.034347 -2.541540 1.752830

C	2.381807	-2.037853	0.653349
N	1.568213	-0.987416	1.069592
C	1.724148	-0.872592	2.379781
N	2.606884	-1.792281	2.832762
C	2.481897	-2.457586	-0.782945
C	3.733985	-1.926649	-1.520739
Cu	0.010924	-0.063906	0.007399
N	-1.478420	-0.082403	1.838295
N	0.532592	1.914710	0.429299
C	-0.483973	2.760066	0.577669
N	-0.030441	4.026814	0.689725
C	1.345818	3.998616	0.606609
C	1.697874	2.680521	0.436810
C	3.097794	2.158845	0.279482
C	3.563736	1.882474	-1.183586
O	2.173134	0.281480	-2.367019
C	3.185803	0.495739	-1.688374
N	4.006821	-0.507615	-1.291240
H	4.654042	1.997162	-1.219794
H	3.234355	1.258890	0.888621
H	3.766624	2.919631	0.696648
H	-1.531800	2.493958	0.564569
H	-0.609065	4.849283	0.803577
H	4.830464	-0.268382	-0.756602
H	4.626290	-2.467953	-1.193517
H	1.579605	-2.126421	-1.297835
H	2.498850	-3.552002	-0.850468
H	1.203125	-0.175395	3.018860
H	2.874147	-1.929805	3.798644
H	3.730118	-3.359791	1.863305
H	1.946708	4.892570	0.681390
H	3.133779	2.620812	-1.865314
H	3.613606	-2.102725	-2.595005
C	-1.715324	0.735866	2.930113
C	-2.259037	-1.143020	2.003079
N	-2.980350	-1.030747	3.148600
C	-2.646705	0.162193	3.757434
H	-2.319456	-1.993230	1.336012
H	-3.643509	-1.713340	3.491959
H	-3.088260	0.482625	4.688953
H	-1.206612	1.680971	3.057704
N	-0.631001	0.652404	-1.833290
C	-1.797417	0.040404	-2.503482
C	-1.635547	-1.484143	-2.583102
C	-1.664372	-2.140379	-1.199922
C	-3.045239	0.592153	-1.774475
C	-5.352503	0.257443	-1.032337
O	-2.600080	-2.888799	-0.897515
O	-0.697621	-1.837743	-0.382818
O	-3.028025	1.764302	-1.374130
N	-4.119478	-0.210411	-1.658572
H	-6.088651	-0.546659	-1.079060
H	-5.184433	0.538347	0.013406
H	-5.736559	1.131502	-1.565234
H	-4.011384	-1.206751	-1.810623

H	-1.867148	0.429148	-3.531522
H	-0.677519	-1.695278	-3.074380
H	-2.419926	-1.919171	-3.208893
H	-0.834840	1.647581	-1.731128
H	0.225089	0.569037	-2.392347

2$^{\mathbf{I}}$(H_2O) - DCu(I) (H2O)_LB , 6-311+g(2df,2p), Energy(B3LYP/SB)= -2248.006212

0 1

C	0.934411	-0.042724	-0.273623
H	0.855305	0.365429	-1.288015
C	0.474285	1.041657	0.729077
C	2.387954	-0.467253	0.009763
H	0.348280	0.587839	1.719184
H	1.253544	1.806836	0.795861
O	2.634263	-1.505244	0.627565
N	3.346648	0.371299	-0.453317
H	3.058088	1.238886	-0.887786
C	4.761537	0.180967	-0.154878
H	5.354453	0.743481	-0.879943
H	5.009303	0.521630	0.857882
H	5.005664	-0.880598	-0.232233
N	0.070091	-1.262484	-0.210249
H	0.287030	-1.852238	-1.015484
H	0.407265	-1.809002	0.590472
C	-0.835757	1.725324	0.251566
O	-1.918921	1.151236	0.664611
O	-0.740656	2.695032	-0.507297
Cu	-1.842253	-0.860180	-0.082085
O	-3.799857	-0.385891	-0.167145
H	-3.555136	0.546466	0.078209
H	-4.248850	-0.354084	-1.026021

2$^{\mathbf{I}}$**(Im)** - DCu(I) (**Im**)_LB , 6-311+g(2df,2p), Energy(B3LYP/SB)= -2397.8328

0 1

C	2.118107	-0.648524	-1.076612
H	2.858758	-1.140709	-1.725496
C	1.877024	0.766964	-1.629839
C	2.717906	-0.790788	0.342236
H	2.849369	1.232415	-1.820725
H	1.361817	0.671114	-2.594088
O	2.720340	-1.917082	0.859213
N	3.271801	0.298398	0.905903
H	2.975649	1.224488	0.573235
C	3.866109	0.219808	2.234224
H	4.358929	1.171115	2.446744
H	3.108358	0.025274	3.003377

H	4.601372	-0.588617	2.267016
N	0.872252	-1.481328	-1.085313
H	0.725231	-1.844675	-2.027617
H	1.077872	-2.291657	-0.491250
C	1.051813	1.680959	-0.688764
O	1.687733	2.512013	-0.005995
O	-0.207541	1.468693	-0.659017
Cu	-0.798867	-0.567842	-0.513822
C	-3.604981	-1.062670	0.432693
C	-4.319360	0.995589	0.848369
C	-3.033148	1.037272	0.383148
H	-3.600752	-2.139610	0.353420
H	-5.554914	-0.724692	1.171796
H	-5.001221	1.772444	1.158289
H	-2.366900	1.871247	0.212219
N	-2.600872	-0.254973	0.127247
N	-4.667681	-0.343721	0.873246

$\mathbf{2^{I}(Im)_2}$ - DCu (I) (**Im**)$_2$ _LB , 6-311+g(2df,2p), Energy(B3LYP/SB)= -2624.077404

0 1

C	-1.415631	-2.186345	-0.507832
H	-1.649399	-3.182828	-0.916693
C	-1.083691	-2.384589	0.984661
C	-2.655104	-1.348029	-0.892929
H	-1.930547	-2.894259	1.457706
H	-0.216669	-3.053571	1.044949
O	-2.885381	-1.174155	-2.097439
N	-3.456927	-0.908900	0.097139
H	-3.047927	-0.822518	1.039700
C	-4.642804	-0.124260	-0.212035
H	-5.212404	0.019215	0.709673
H	-4.384244	0.855314	-0.635311
H	-5.261622	-0.652987	-0.942472
N	-0.266159	-1.611259	-1.262397
H	0.449633	-2.329521	-1.371486
H	-0.616485	-1.401641	-2.200346
C	-0.762478	-1.081765	1.751240
O	-1.735198	-0.467978	2.274123
O	0.446780	-0.701072	1.747525
Cu	0.750699	0.070237	-0.440649
C	-0.722030	2.218115	0.903435
C	-0.672827	4.060821	-0.339724
C	-0.016128	3.041123	-0.977784
H	-0.954422	1.535198	1.714570
H	-1.644403	3.995304	1.568278
H	-0.861620	5.087408	-0.614552

H	0.474602	3.051276	-1.940037
N	-0.051945	1.900788	-0.196774
N	-1.114127	3.518279	0.852500
H	5.243654	-1.063681	1.504902
H	6.053694	-0.429322	-0.839174
H	3.726829	0.208207	-2.206036
N	2.780288	-0.278904	-0.333109
C	5.020247	-0.428415	-0.527228
C	3.856984	-0.118909	-1.184434
N	4.631990	-0.782340	0.751203
C	3.278174	-0.679002	0.827084
H	2.681320	-0.888944	1.704813

6^{I} - LCu(I) _LB , 6-311+g(2df,2p), Energy(B3LYP/SB)= -2417.46767

1 1

6	4.033387	-0.334136	-0.032800
6	2.750578	-0.033986	-0.417571
7	1.903193	-1.002693	0.122544
6	2.661844	-1.852632	0.810139
7	3.954546	-1.476310	0.738679
6	2.256923	1.086683	-1.281432
6	1.842032	2.381001	-0.545601
29	0.017079	-1.041056	-0.043849
7	-1.873988	-1.020091	-0.127155
6	-2.662511	-1.716539	-0.939446
7	-3.943503	-1.326425	-0.781782
6	-3.982170	-0.332972	0.175536
6	-2.687242	-0.137080	0.580541
6	-2.140450	0.827286	1.590945
6	-1.654635	2.191006	1.025474
8	-0.822280	1.812493	-1.207337
6	-0.562456	2.075540	-0.033728
7	0.728113	2.240088	0.395533
1	-1.323327	2.806794	1.870228
1	-1.320642	0.342169	2.136386
1	-2.919022	1.038210	2.332225
1	-2.347456	-2.486759	-1.627685
1	-4.739206	-1.706162	-1.278932
1	0.876079	2.565548	1.341648
1	2.689690	2.777166	0.020943
1	1.411513	0.742929	-1.885118
1	3.051809	1.363965	-1.984134
1	2.316388	-2.723772	1.347032
1	4.733629	-1.964955	1.161791
1	4.975233	0.147142	-0.249134
1	-4.905784	0.137188	0.477271
1	-2.495084	2.704986	0.549305
1	1.568794	3.119239	-1.308574

6^{I} (**Im**) - LCu(I) (**Im**) _LB , 6-311+g(2df,2p), Energy(B3LYP/SB)= -2643.71497

1 1

C	-0.577374	3.949008	0.082486
C	-0.666903	2.664660	-0.399591
N	0.233734	1.868476	0.304869
C	0.842746	2.659102	1.181388
N	0.375290	3.924160	1.080774
C	-1.550162	2.125427	-1.486087
C	-3.033706	1.964732	-1.084747
Cu	0.660067	-0.058582	0.089352
N	-0.086000	-1.886399	-0.000878
C	0.377152	-2.823925	-0.819702
N	-0.347763	-3.957762	-0.694920
C	-1.325653	-3.738974	0.252503
C	-1.165135	-2.443948	0.677049
C	-1.990920	-1.662741	1.655743
C	-3.351639	-1.167918	1.093555
O	-2.983250	-0.819304	-1.264950
C	-3.187711	-0.318038	-0.160985
N	-3.240658	1.035555	0.018641
H	-3.876302	-0.619334	1.885453
H	-1.401497	-0.803941	1.998696
H	-2.196865	-2.277284	2.540010
H	1.212523	-2.716549	-1.495883
H	-0.207268	-4.810893	-1.220106
H	-3.421587	1.404567	0.941619
H	-3.459963	2.929390	-0.792400
H	-1.171870	1.149525	-1.806236
H	-1.509195	2.787812	-2.360212
H	1.610266	2.358226	1.879422
H	0.684921	4.715198	1.630438
H	-1.085638	4.858869	-0.200135
H	-2.043018	-4.499590	0.521292
H	-3.970369	-2.028247	0.820554
H	-3.589449	1.596433	-1.952450
C	3.580922	-1.074114	0.523463
C	4.921321	0.229858	-0.663941
C	3.616742	0.616011	-0.827907
H	3.263506	-1.862959	1.190727
H	5.676650	-1.369304	0.537810
H	5.846988	0.606353	-1.072188
H	3.221480	1.420050	-1.431616
N	2.786537	-0.204015	-0.083503
N	4.879238	-0.845708	0.199909

6$^{\mathbf{I}}$(**Im**)($H_{14}H_{13}$) - LCu(I) (**Im**) _LB , 6-311+g(2df,2p), Energy(B3LYP/SB)= -2643.70101

1 1

C	-1.62536	3.56643	0.14306
C	-1.67028	2.19973	0.25325
N	-2.96317	1.78509	-0.07264
C	-3.66706	2.87861	-0.36642
N	-2.88641	3.96965	-0.24771
C	-0.56323	1.25675	0.60832
C	0.29223	0.84339	-0.61639
Cu	-3.6278	0.0171	-0.03888

N	6.13579	-1.81575	-0.42241
C	7.34808	-2.27246	-0.18634
N	8.20086	-1.26687	0.16688
C	7.47587	-0.09062	0.15389
C	6.19968	-0.44904	-0.21534
C	4.99138	0.41879	-0.40248
C	3.83321	0.00356	0.5344
O	2.52753	2.0394	0.4628
C	2.58587	0.82516	0.26841
N	1.50243	0.14073	-0.22747
H	4.1306	0.17548	1.57535
H	4.65144	0.34418	-1.44491
H	5.24426	1.46853	-0.22233
H	7.66268	-3.30516	-0.25638
H	9.18153	-1.36288	0.39258
H	1.59374	-0.84623	-0.42435
H	-0.2804	0.19848	-1.29136
H	-0.97258	0.36378	1.09766
H	0.10301	1.74543	1.32471
H	-4.70863	2.90959	-0.65008
H	-3.18289	4.92435	-0.40789
H	-0.81839	4.26449	0.30809
H	7.92195	0.86309	0.39552
H	3.64369	-1.069	0.41673
H	0.57597	1.74579	-1.16834
C	-4.57346	-2.54699	-1.01049
C	-5.08899	-3.69901	0.81261
C	-4.62414	-2.4636	1.1686
H	-4.44096	-2.30454	-2.05444
H	-5.32838	-4.50672	-1.15237
H	-5.43562	-4.53501	1.40034
H	-4.49976	-2.04277	2.15478
N	-4.30364	-1.74976	0.0214
N	-5.04845	-3.72971	-0.56646

7^{I} - LCu(I) (NMA)_LB , 6-311+g(2df,2p), Energy(B3LYP/SB)= -2666.02238

1 1

C	-1.121143	4.036410	-0.146750
C	-1.321096	2.712259	-0.450095
N	-0.145924	2.020783	-0.159286
C	0.729826	2.906141	0.304144
N	0.172538	4.135688	0.328985
C	-2.540630	2.047201	-1.013570
C	-3.575522	1.556074	0.023721
Cu	0.190171	0.143141	-0.337596
N	0.042551	-1.750360	-0.604449
C	0.396627	-2.442266	-1.681056
N	0.042327	-3.738025	-1.537548
C	-0.572995	-3.885108	-0.311590
C	-0.578521	-2.641218	0.266876
C	-1.161191	-2.198693	1.576525
C	-2.675240	-1.853053	1.534945

O	-3.243536	-1.124200	-0.692847
C	-3.041546	-0.813238	0.479029
N	-3.103197	0.487060	0.901251
H	-2.978119	-1.526745	2.537574
H	-0.595791	-1.328899	1.933783
H	-1.021880	-2.989710	2.322393
H	0.896444	-2.048911	-2.553826
H	0.190164	-4.468823	-2.221529
H	-3.016185	0.681344	1.889346
H	-3.894240	2.386044	0.661529
H	-2.241804	1.196830	-1.634711
H	-3.057898	2.754661	-1.673344
H	1.741461	2.675487	0.602725
H	0.634510	4.985717	0.624667
H	-1.765509	4.897747	-0.239354
H	-0.955924	-4.835799	0.027280
H	-3.246454	-2.754049	1.292563
H	-4.451123	1.190179	-0.523223
C	3.557017	-0.228766	0.651487
C	5.119330	1.122603	-0.704028
H	5.558701	-0.581466	0.500844
H	6.110601	0.959660	-1.131521
N	4.801140	0.005096	0.178649
O	2.594387	0.492380	0.334815
H	5.117472	2.073184	-0.158379
H	4.383965	1.180681	-1.510188
C	3.390592	-1.410982	1.584271
H	4.311369	-1.982096	1.735783
H	3.037051	-1.050352	2.556078
H	2.622320	-2.077102	1.179367

$8a^I$ - LDCu(I) _LB , 6-311+g(2df,2p), Energy(B3LYP/SB)= -2948.82952

0 1

C	4.460217	-0.567429	0.359304
C	4.199198	-1.911065	0.520839
N	5.335843	-2.652508	0.245651
C	6.267530	-1.776076	-0.067554
N	5.787205	-0.497924	-0.016218
C	2.898954	-2.569063	0.875172
C	2.084376	-2.917076	-0.387575
Cu	-1.801418	0.411623	-0.626757
N	-3.380682	-0.694372	-0.399712
C	-4.488192	-0.068976	-0.019600
N	-5.453513	-0.971866	0.271972
C	-4.933916	-2.238249	0.073662
C	-3.636098	-2.057296	-0.340603
C	-2.623182	-3.111608	-0.678976
C	-1.613846	-3.480322	0.459336
O	-0.170540	-1.543243	0.555917
C	-0.281808	-2.751040	0.327776
N	0.770366	-3.498055	-0.107088

H	-1.451941	-4.565075	0.450519
H	-2.071622	-2.803906	-1.575505
H	-3.170826	-4.021714	-0.948680
H	-4.570889	1.007727	0.073942
H	-6.386380	-0.748933	0.590512
H	0.641013	-4.491008	-0.242155
H	1.933043	-2.008794	-0.979850
H	2.297275	-1.906480	1.504774
H	3.099393	-3.484969	1.443691
H	7.292438	-2.004557	-0.328373
H	6.304941	0.349162	-0.204548
H	3.841676	0.315761	0.447289
H	-5.517591	-3.131814	0.237915
H	-2.042744	-3.223108	1.432815
H	2.636905	-3.631374	-1.005310
O	-2.701161	2.283464	-0.020798
C	-2.121045	2.957854	0.903615
O	-2.660355	3.636480	1.788624
C	-0.560819	2.902289	0.935090
H	-0.211810	3.863598	1.325619
H	-0.257299	2.147844	1.675047
N	0.039398	1.158242	-0.754622
H	0.488237	0.997427	-1.656927
H	0.593512	0.612114	-0.089864
C	0.133878	2.595126	-0.410191
H	-0.385553	3.146358	-1.202961
C	1.607664	3.033420	-0.363756
N	1.827865	4.362608	-0.539980
O	2.527869	2.232030	-0.173391
H	1.027268	4.976194	-0.616099
C	3.147233	4.960531	-0.386248
H	3.483688	4.930865	0.657289
H	3.873757	4.418876	-0.997981
H	3.101878	5.999898	-0.719773

8b^{I} - LDCu (I) _LB , 6-311+g(2df,2p), Energy(B3LYP/SB)= -2948.83683

0 1

C	-2.299290	-3.665390	-1.446230
C	-2.058234	-2.730127	-0.467857
N	-1.099033	-1.836516	-0.932368
C	-0.772901	-2.226262	-2.157705
N	-1.478108	-3.330345	-2.506592
C	-2.700243	-2.635736	0.882936
C	-3.705446	-1.472215	1.046078
Cu	-0.129068	-0.175032	-0.240204
N	-1.253776	1.690491	-0.678716
C	-0.794355	2.149663	-1.838579

N	-0.955699	3.488582	-1.922230
C	-1.544544	3.922008	-0.749954
C	-1.738044	2.796706	0.015298
C	-2.434659	2.732383	1.351181
C	-1.936506	1.664666	2.353501
O	-2.896829	-0.266164	3.424441
C	-2.677116	0.325864	2.365637
N	-3.082497	-0.162752	1.156936
H	-2.024737	2.045086	3.374642
H	-3.514145	2.593743	1.196068
H	-2.329155	3.718807	1.817680
H	-0.278049	1.563165	-2.585906
H	-0.587387	4.065497	-2.666569
H	-2.708790	0.268170	0.316539
H	-4.401351	-1.454652	0.199497
H	-1.937604	-2.543480	1.665700
H	-3.236966	-3.574096	1.072831
H	-0.035698	-1.732354	-2.777217
H	-1.401403	-3.823769	-3.385047
H	-2.960099	-4.519060	-1.475049
H	-1.780697	4.960829	-0.572207
H	-0.871884	1.457051	2.184994
H	-4.279395	-1.629960	1.962390
O	1.239560	-0.129968	-2.052332
C	2.085644	0.827169	-1.957955
O	2.192988	1.806169	-2.718703
C	3.076546	0.772792	-0.761821
H	4.075964	0.599987	-1.181051
H	3.119551	1.768347	-0.300733
N	1.509000	0.025037	1.015770
H	1.425647	-0.599691	1.819048
H	1.618543	0.948088	1.440917
C	2.774461	-0.290145	0.307660
H	2.629107	-1.253709	-0.193144
C	3.931971	-0.390509	1.317173
N	5.015688	-1.101441	0.897717
O	3.868964	0.138325	2.427858
H	5.019447	-1.455235	-0.050010
C	6.244595	-1.191928	1.673912
H	6.922711	-0.354850	1.463184
H	5.989669	-1.171560	2.735128
H	6.751913	-2.132365	1.440832

Supporting Information

TableS2. Primary properties calculated at the B3LYP/SB optimized structures of all species: SB = 6-31+G(d); LB = 6-311+(2df,2p)

Compound	E(B3LYP/SB)[a]	E(B3LYP/LB)[a]	ZPE[a]	H_0[a]	S_{298}[b]	*N*[c]	ΔG_{solv}[d]	E(MP2/SB)[a]	E('MP2/LB')[a,e]
H_2O	-76.42257	-76.46243	0.02110	0.02487	45.1	1	-3.9	-76.20977	-76.24963
H^+(aq)	0.00000	0.00000	0.00000	0.00236	26.0	1	-264.6	0.0000	0.0000
Im	-226.22693	-226.29723	0.07115	0.07587	65.3	1	-10.1	-1943.32147	-225.60587
$Cu(H_2O)_5^{2+}$	-2021.91938	-2022.25692	0.12339	0.13923	118.7	1	-228.6	-2019.58636	-2019.92390
Cu(**Im**)$(H_2O)_4^{2+}$	-2171.78282	-2172.15278	0.17263	0.19056	132.2	2	-198.8	-2168.97044	-2169.34040
Cu(**Im**)$_2(H_2O)_3^{2+}$-trans	-2321.63504	-2322.03784	0.22188	0.24189	156.4	4	-175.6	-2318.34791	-2318.75072
Cu(**Im**)$_2(H_2O)_3^{2+}$-cis	-2321.63227	-2322.03455	0.22201	0.24197	145.9	8	-175.6	-2318.34556	-2318.74783
NMA	-248.53733	-248.62030	0.10213	0.10979	81.3	1	-9.3	-247.74753	-247.83050
$Cu(H_2O)_3^+$	-1869.45158	-1869.71710	0.07266	0.08376	97.7	1	-75.0	-1867.54591	-1867.81143
Cu(**Im**)$(H_2O)^+$	-1942.85198	-1943.11320	0.09833	0.10778	92.4	1	-45.0	-1940.68264	-1940.94386
Cu(**Im**)$_2^+$	-2092.69470	-2092.98826	0.14775	0.15902	105.7	1	-45.7	-2090.0525	-2090.34612

								6	
Cu(**Im**)$(H_{13}H_{14})^+$	-2643.70101	-2644.16174	0.34082	0.36471	180.3	288	-57.7	-2639.37273	-2639.83346
**1** - DH (Asp1)	-531.75258	-531.92889	0.16384	0.17568	104.1	1	-43.0	-530.19025	-530.36656
**2a**$(H_2O)_2$ - DCu(II)$(H_2O)_2^+$	-2324.21365	-2324.61257	0.20576	0.22398	135.5	1	-88.4	-2320.94593	-2321.34485
**2b**$(H_2O)_2$ - DCu(II)$(H_2O)_2^+$	-2324.20127	-2324.60095	0.20497	0.22370	140.1	2	-91.5	-2320.92479	-2321.32447
**2c**$(H_2O)_2$ - DCu(II)$(H_2O)_2^+$	-2324.18033	-2324.58006	0.20469	0.22289	135.6	1	-82.8	-2320.91112	-2321.31086
**2a**(**Im**)(H_2O) - DCu(II) (**Im**) $(H_2O)^+$	-2474.04439	-2474.47677	0.25516	0.27524	147.7	2	-76.2	-2470.30121	-2470.73358
**2b**(**Im**)(H_2O) - DCu(II) (**Im**) $(H_2O)^+$	-2474.04747	-2474.47958	0.25411	0.27475	151.1	2	-73.1	-2470.30877	-2470.74089
**2a**$(\mathbf{Im})_2$ - DCu(II) $(\mathbf{Im})_2^+$	-2623.87320	-2624.33810	0.30265	0.32571	165.0	4	-66.0	-2619.66463	-2620.12967
**2b**$(\mathbf{Im})_2$ - DCu(II) $(\mathbf{Im})_2^+$	-2623.86536	-2624.33053	0.30301	0.32580	161.6	4	-64.2	-2619.66477	-2620.12980
**3 - Asp1Ala2**	-779.08983	-776.78361	0.24682	0.26461	137.7	8	-49.1	-776.78361	-777.03836
**4**(H_2O) - D1A2_Cu2_W	-2495.12810	-2491.32841	0.26472	0.28583	150.7	2	-66.9	-2491.32841	-2491.77117
4$(H_2O)(CO_2^-)$ - D1A2_Cu2_W_Om	-2494.72331	-2490.92930	0.25074	0.27177	150.8	2	-30.6	-2490.92930	-2491.36608

**4**(Im) - D1A2_Cu2_Im	-2644.96233	-2640.68527	0.31350	0.33691	165.6	8	-60.0	-2640.68527	-2641.16171
**4**(**Im**)(CO_2^-) - D1A2_Cu2_Im_Om	-2644.53791	-2640.27250	0.29849	0.32229	167.5	4	-32.2	-2640.27250	-2640.74279
**4**(**Im**)$_2$ - D1A2_Cu2_Im2	-2871.21014	-2866.26446	0.38560	0.41482	197.1	4	-54.8	-2866.26446	-2866.80980
**4**(**Im**)$_2$(CO_2^-) - D1A2_Cu2_Im2_Om	-2870.76150	-2865.81938	0.37144	0.40041	198.8	4	-42.1	-2865.81938	-2866.35829
**5** - L	-777.24067	-777.47732	0.26584	0.28220	131.9	63	-18.0	-774.86290	-775.09954
**6**(H_2O)$_2$ - L Cu(II) (H_2O)$_2^{2+}$	*-2569.99388*	*-2570.45670*	0.31979	0.34282	155.6	8	-164.5	-2565.92223	-2566.38506
**6**(**Im**)(H_2O) - Cu(II)(**Im**)(H_2O)$^+$	*-2719.83236*	*-2720.32831*	0.36834	0.39372	169.4	4	-146.8	-2715.29169	-2715.78764
**7** - LCu(II)(**Im**)(NMA)	-2891.95984	-2892.50096	0.44790	0.47839	201.3	8	-131.8	-2886.85327	-2887.39439
**8**a - LDCu(II)$^+$	-2948.63962	-2949.20121	0.42446	0.45393	192.7	6	-63.1	-2943.44125	-2944.00284
**8**b - LDCu(II)$^+$	-2948.63756	-2949.19998	0.42416	0.45404	201.0	16	-69.8	-2943.41793	-2943.98035
**9** - LDCu(II) (**Im**)$^+$	-3174.89160	-3175.52199	0.49831	0.53271	214.7	6	-51.3	-3169.03127	-3169.66142
**2**^{I}(H_2O) - DCu(I) (H2O)	-2248.00621	-2248.36998	0.17988	0.19569	126.3	8	-32.9	-2244.97253	-2245.33630

**2**^{I}(**Im**) - DCu(I) (**Im**)	-2397.83280	-2398.22866	0.22848	0.24641	137.5	2	-27.4	-2394.32995	-2394.72582
**2**^{I}(**Im**)$_2$ - DCu (I) (**Im**)$_2$	-2624.07740	-2624.54246	0.30031	0.32402	168.1	4	-22.9	-2619.89564	-2620.36070
**6**^{I} - LCu(I)	-2417.46767	-2417.85754	0.26940	0.28688	132.5	2	-50.9	-2413.83501	-2414.22487
**6**^{I} (**Im**) - LCu(I) (**Im**)	-2643.71497	-2644.17485	0.34102	0.36448	167.7	4	-47.5	-2639.40084	-2639.86072
**6**^{I}(**Im**)($H_{14}H_{13}$) - LCu(I) (**Im**)	-2643.70101	-2644.16174	0.34082	0.36471	180.3	288	-57.7	-2639.37273	-2639.83346
**7**^{I} - LCu(I) (NMA)	-2666.02238	-2666.49446	0.37229	0.39861	183.9	8	-46.2	-2661.60777	-2662.07985
**8a**^{I} - LDCu(I)	-2948.82952	-2949.39180	0.42065	0.45181	212.3	16	-39.9	-2943.64281	-2944.20508
**8b**^{I} - LDCu (I)	-2948.83683	-2949.39836	0.42107	0.45089	194.9	6	-33.3	-2943.66270	-2944.22422

[a] Hartrees

[b] cal/mol-K

[c] Number of conformations (assumed equally populated)

[d] kcal/mol

[e] See text for definition of 'MP2/LB'

Table S3. Relative energies at 298 K of species discussed in the text: enthalpies based on B3LYP/LB energies

Process	Eqn No.	$\Delta H_{(g)}$ kJ mol^{-1}	$-T\Delta S_{(g)}$ kJ mol^{-1}	$\Delta G_{(g)}$ kJ mol^{-1}	$\Delta\Delta G_{solv}$ kJ.mol^{-1}	$\Delta G_{(aq)}$ kJ mol^{-1}
**1**+ $Cu(H_2O)_5^{2+}$ → **2a**$(H_2O)_2$ + $3H_2O$ + H^+	(4a)	452.5	-68.6	383.9	-390.2	-46.3
**1**+ $Cu(H_2O)_5^{2+}$ → **2b**$(H_2O)_2$ + $3H_2O$ + H^+	(4b)	482.3	-76.1	406.3	-403.2	-37.0
**1** + $Cu(H_2O)_5^{2+}$ → **2c**$(H_2O)_2$ + $3H_2O$ + H^+	(4c)	490.6	-68.8	421.8	-381.4	0.5
**3** + $Cu(H_2O)_5$ → **4**(H_2O) + 4 H_2O+ H^+	(5)	433.6	-90.6	343.0	-323.4	-20.4
**Im** + $Cu(H_2O)_5^{2+}$ → Cu(**Im**)$(H_2O)_4^{2+}$ + H_2O	(6a)	-159.4	6.5	-152.9	150.3	-2.6
2 **Im** + $Cu(H_2O)_5^{2+}$ → Cu(**Im**)$_2(H_2O)_3^{2+}$ + 2 H_2O	(6b)	-290.5	-0.3	-290.8	273.2	-17.6
**2a**$(H_2O)_2$ + **Im** → **2a**(H_2O) + H_2O	(7a)	-85.1	3.9	-81.2	77.0	-4.2
**1** + Cu(**Im**)$(H_2O)_4^{2+}$ → **2a**(**Im**)(H_2O) + 3 H_2O + H^+	(7b)	526.8	-71.2	455.6	-463.5	-47.9
**2a**(**Im**)(H_2O) + **Im** → **2a**(**Im**)$_2$ + H_2O	(8a)	-62.3	6.1	-56.2	68.4	12.2
**1** + Cu(**Im**)$_2(H_2O)_3^{2+}$ → **2a**(**Im**)$_2$ + 3 H_2O + H^+	(8b)	595.6	-58.4	537.2	-517.9	-20.7
**4**(H_2O) + **Im** → **4**(**Im**) + H_2O	(9a)	-86.7	3.1	-83.6	66.2	-17.4
**3** + $Cu(H_2O)_5^{2+}$ + **Im** → **4**(**Im**) + $5H_2O$ + H^+	(9b)	347.0	-87.5	259.5	-257.3	-37.8
**4**(**Im**) + **Im** → **4**(**Im**)$_2$	(10)	-45.9	35.9	-10.0	76.6	66.6
**4**(**Im**) + **Im** + **1** → **2a**(**Im**)$_2$ + **3**	(11)	-41.9	28.8	-13.0	12.5	-0.5

**5** + $Cu(H_2O)_5^{2+}$ $\rightarrow$ **6**$(H_2O)_2$ + $3H_2O$	(12)	-298.4	-29.5	-327.9	293.8	-34.0
**6**$(H_2O)_2$ + **Im** $\rightarrow$ **6**(**Im**)(H_2O) + H_2O	(13)	-96.8	9.6	-87.2	99.7	12.5
**6**(**Im**)(H_2O) + NMA $\rightarrow$ **7** + H_2O	(14)	39.4	-3.7	35.7	-85.4	-49.7
**1** + **6**$(H_2O)_2$ $\rightarrow$ 8**a** + $2H_2O$ + H^+	(15a)	649.5	-44.8	604.7	-535.6	29.0
**2a**$(H_2O)_2$ + **5** $\rightarrow$ **8a** + $2H_2O$	(15b)	-101.4	-5.7	-107.1	148.4	41.3
**1** + **6**(**Im**)(H_2O) $\rightarrow$ **9** + H_2O + H^+	(16a)	685.8	-8.4	683.6	-543.8	99.7
**9** $\rightarrow$ **8a** + **Im**	(16b)	54.3	-46.0	8.3	-91.5	-83.2
**9** + H_2O + H^+ $\rightarrow$ **5** + **3a**(H_2O)	(16c)	-70.6	36.4	-34.2	162.9	88.75
**1** + $Cu(H_2O)_3^+$ $\rightarrow$ **2**^{I}(H_2O) + $2H_2O$ + H^+	(17)	892.1	-40.1	852.0	-783.9	28.1
**Im** + $Cu(H_2O)_3^+$ $\rightarrow$ Cu(**Im**)$(H_2O)^+$ + $2H_2O$	(18)	-67.7	-16.6	-84.3	73.5	-10.8
**Im** + Cu(**Im**)$(H_2O)^+$ $\rightarrow$ Cu(**Im**)$_2^+$ + H_2O	(19a)	-105.0	8.5	-96.5	84.2	-12.3
**5** + Cu(**Im**)$(H_2O)^+$ $\rightarrow$ Cu(**Im**)$(H_{13}H_{14})^+$ + H_2O	(19b)	-89.3	-5.2	-94.5	67.0	-27.5
**5** + Cu(**Im**)$(H_2O)^+$ $\rightarrow$ Cu(**Im**)$(H_{14}H_{13})^+$ + H_2O	(19c)	-115.9	2.7	-113.2	102.2	-11.0
**1** + Cu(**Im**)$(H_2O)^+$ $\rightarrow$ **2**^{I}(**Im**) + H_2O + H^+	(20)	896.4	-8.9	887.5	-808.5	39.1
**2**^{I}(**Im**) + **Im** $\rightarrow$ **2**^{I}(**Im**)$_2$	(21)	-39.0	33.6	-5.4	60.8	55.4
**1** + Cu(**Im**)$_2^+$ $\rightarrow$ **2**^{I}(**Im**)$_2$ + H^+	(22)	962.5	16.2	978.7	-831.9	106.8
**5** + $Cu(H_2O)_3^+$ $\rightarrow$ **6**^{I} + 3 H_2O	(23)	-143.8	-23.4	-167.2	126.9	-40.3

**1** + **6**^{I} → **8a**^{I} + H^+	(24a)	1014.7	-3.7	1011.0	-881.3	89.77
**1** + **6**^{I} → **8b**^{I} + H^+	(24b)	995.1	21.4	1016.5	-853.9	122.63
NMA + **6**^{I} → **7**^{I}	(25)	-38.6	26.0	-12.6	58.2	45.6
**Im** + **6**^{I} → **6**^{I}(**Im**)	(26)	-48.2	27.9	-20.3	56.4	36.1
**2a**$(H_2O)_2$ + e^- → **2a**^{I}(H_2O) + H_2O	(27)	-585.9	-42.0	-628.0	215.9	-412.1 [b]
**2a**(**Im**)(H_2O) + e^- → **2**^{I}(**Im**) + H_2O	(28)	-564.2	-31.4	-595.6	187.8	-407.8 [b]
**2a**(**Im**)$_2$ + **5** + e^- + H^+ → **1** + Cu(**Im**)$(H_{13}H_{14})^+$	(29a)	-1487.6	-33.8	-1521.4	994.8	-526.6 [b]
**2a**(**Im**)$_2$ + **5** + e^- H^+ → **1** + Cu(**Im**)$(H_{14}H_{13})^+$	(29b)	-1514.2	-25.9	-1540.1	1030.0	-510.1 [b]
**2a**(**Im**)$_2$ + **5** + e^- + H^+ → **1** + **6**^{I} (**Im**) + **Im**	(30)	-1522.6	-7.4	-1530.1	1037.7	-492.4 [b]
**2a**(**Im**)$_2$ + **5** + e^- + H^+ → **1** + **6**^{I} + 2 **Im**	(31)	-1474.5	-35.3	-1509.8	981.3	-528.5 [b]
**6**$(H_2O)_2$ + e^- → **6**^{I} + 2 H_2O	(32)	-871.0	-64.5	-1540.2	978.3	-561.8 [b]
**6**(**Im**)(H_2O) + e^- → **6**^{I} + **Im** + H_2O	(33)	-774.2	-74.1	-848.3	343.0	-505.3 [b]
**7** + e^- → **6**^{I} + **Im** + NMA	(34)	-734.8	-77.8	-812.6	257.6	-555.0 [b]
**6**(**Im**)(H_2O) + e^- → **6**^{I}(**Im**) + H_2O	(35)	-822.3	-46.2	-868.6	399.4	-469.2 [b]

[a] Numbered structures are presented in Figures 1 – 4. [b] The enthalpy correction, 50 kJ mol^{-1} has not been added
